# Predicting the onset of preeclampsia by longitudinal monitoring of metabolic changes throughout pregnancy with Raman spectroscopy

**DOI:** 10.1002/btm2.10595

**Published:** 2023-08-31

**Authors:** Saman Ghazvini, Saji Uthaman, Lilly Synan, Eugene C. Lin, Soumik Sarkar, Mark K. Santillan, Donna A. Santillan, Rizia Bardhan

**Affiliations:** ^1^ Department of Chemical and Biological Engineering Iowa State University Ames Iowa USA; ^2^ Nanovaccine Institute Iowa State University Ames Iowa USA; ^3^ Department of Chemistry and Biochemistry National Chung Cheng University Chiayi Taiwan; ^4^ Department of Mechanical Engineering Iowa state University Ames Iowa USA; ^5^ Department of Obstetrics and Gynecology, Carver College of Medicine University of Iowa, Hospitals & Clinics Iowa City Iowa USA

**Keywords:** metabolism, patient, preeclampsia, pregnancy, Raman spectroscopy

## Abstract

Preeclampsia is a life‐threatening pregnancy disorder. Current clinical assays cannot predict the onset of preeclampsia until the late 2nd trimester, which often leads to poor maternal and neonatal outcomes. Here we show that Raman spectroscopy combined with machine learning in pregnant patient plasma enables rapid, highly sensitive maternal metabolome screening that predicts preeclampsia as early as the 1st trimester with >82% accuracy. We identified 12, 15 and 17 statistically significant metabolites in the 1st, 2nd and 3rd trimesters, respectively. Metabolic pathway analysis shows multiple pathways corresponding to amino acids, fatty acids, retinol, and sugars are enriched in the preeclamptic cohort relative to a healthy pregnancy. Leveraging Pearson's correlation analysis, we show *for the first time* with Raman Spectroscopy that metabolites are associated with several clinical factors, including patients' body mass index, gestational age at delivery, history of preeclampsia, and severity of preeclampsia. We also show that protein quantification alone of proinflammatory cytokines and clinically relevant angiogenic markers are inadequate in identifying at‐risk patients. Our findings demonstrate that Raman spectroscopy is a powerful tool that may complement current clinical assays in early diagnosis and in the prognosis of the severity of preeclampsia to ultimately enable comprehensive prenatal care for all patients.

**Translational Impact Statement**

Here we have used Raman spectroscopy as a translationally relevant tool to longitudinally track metabolic reprogramming in pregnant patient plasma. Through this approach we have identified patients with preeclampsia, a disorder with poor maternal and neonatal outcome, in the first trimester with >82% accuracy that is not achievable with current clinical tools. Our correlation analysis show association of metabolites to severity of preeclampsia in all three trimesters suggesting that metabolic screening throughout pregnancy will complement prenatal care and reveal mechanisms associated with the onset of preeclampsia.

## 1 INTRODUCTION

Preeclampsia is a hypertensive disorder that impacts 10% of pregnancies globally, and is characterized by the new onset of hypertension (blood pressure above 140/90 mmHg) after 20 weeks of gestation and by proteinuria (protein in the urine).^1^ Preeclampsia is a major contributor to maternal morbidity and is associated with adverse fetal outcomes, including placental abruption, fetal growth restriction, preterm birth, and perinatal death.^2^ Despite its prevalence, current clinical measures are inadequate in predicting the onset or the severity of preeclampsia including the HELLP (hemolysis, elevated liver enzymes, and low platelet count) syndrome, a life‐threatening form of preeclampsia.^3^ Many clinical risk prediction models have been applied to predict preeclampsia based on a patient's history of hypertension, previous chronic hypertension, hypertension in current pregnancy, and preeclampsia in a previous pregnancy. However, the sensitivity of these models has been low with high false positive rates and only 58%–68% accuracy.^4^, ^5^ Clinical tests for liver and kidney function that assess plasma levels of liver enzymes during the first 20 weeks of pregnancy have also been considered a risk factor; however, a predictive cut‐off value was not established to implement in clinical practice.^6^ Pro‐inflammatory cytokines have also been correlated to the risk of preeclampsia. However, inter‐ and intra‐assay discrepancies between studies due to the variability in the type of kit used, the type of sample studied, and the timing of sample collection have rendered these protein‐based assays unreliable as a predictive tool.^7^ The ratio of maternal angiogenic proteins including soluble Fms‐like tyrosine kinase 1(sFlt‐1) and placental growth factor (PlGF) provides a measure of anti‐angiogenesis, and is measured in the mid‐to‐late 2nd trimester to predict preeclampsia.^8^, ^9^ Whereas sFlt‐1/PlGF ratio has shown high predictive power‐area under the curve (AUC) of 83%–91%—in the late 2nd and 3rd trimester,^10^, ^11^, ^12^ in early pregnancy the accuracy of these markers remain poor and fail to identify at risk patients.^13^ There is a critical need for (i) innovative methodologies that enable early, accurate, and affordable screening for patients, (ii) are complementary to current prenatal care to seamlessly fit into the clinical workflow, and (iii) establish novel clinically significant markers with high sensitivity and specificity to not only allow risk prediction but also identify mechanisms that lead to preeclampsia.

Literature findings show that dynamic metabolic changes throughout pregnancy control placental function, nutrient transport at the maternal–fetal interface, and fetal development.^14^ Uterine abnormalities can rewire the placental metabolome resulting in oxidative stress, the release of soluble toxic factors in the bloodstream that lead to inflammatory response, and endothelial dysfunction; each of these factors has been associated with preeclampsia.^15^, ^16^, ^17^ Mass spectrometry is widely accepted in metabolic profiling enabling the highly precise identity of metabolites.^18^ However, it is also cost‐ and time‐intensive, require a multi‐step extraction process that is prone to errors, and it is destructive, that is, the sample is consumed during the measurement and cannot be re‐measured or archived. Raman spectroscopy (RS) is an emerging optical metabolic technique where biomolecules in a sample interact with incident light and induce vibrations that results in inelastic light scattering of incident photons.^19^, ^20^, ^21^ The differences in the incidence frequency and molecular vibrational frequencies manifest as a biochemical “fingerprint” of that sample. Raman spectroscopy offers multiple merits as an accurate tool for metabolic profiling throughout pregnancy. These include: (i) Raman enables rapid (~30 min) measurement of metabolites in each sample; (ii) it requires minimal sample volumes (~3 μL) while allowing multiplexing in a high‐throughput format; (iii) Raman is a label‐free and extraction‐free approach where biofluids and tissues can be directly measured without further preparation; and (iv) it is a non‐destructive approach, that is samples can be stored for future analysis. Our group has leveraged these merits of Raman spectroscopy for metabolic, and biomarker analysis in cells, tissues, and biofluids.^22^, ^23^, ^24^, ^25^, ^26^, ^27^ This study also builds upon our recent work where we showed metabolite changes in sera measured with Raman when combined with clinical information of pregnant patients who delivered preterm enables an unprecedented 87.6% accuracy of risk stratification in the first trimester.^28^ Indeed other groups have also leveraged Raman spectroscopy for in vivo measure of metabolic changes in pregnant patient cervix.^29^, ^30^

**SCHEME 1:**
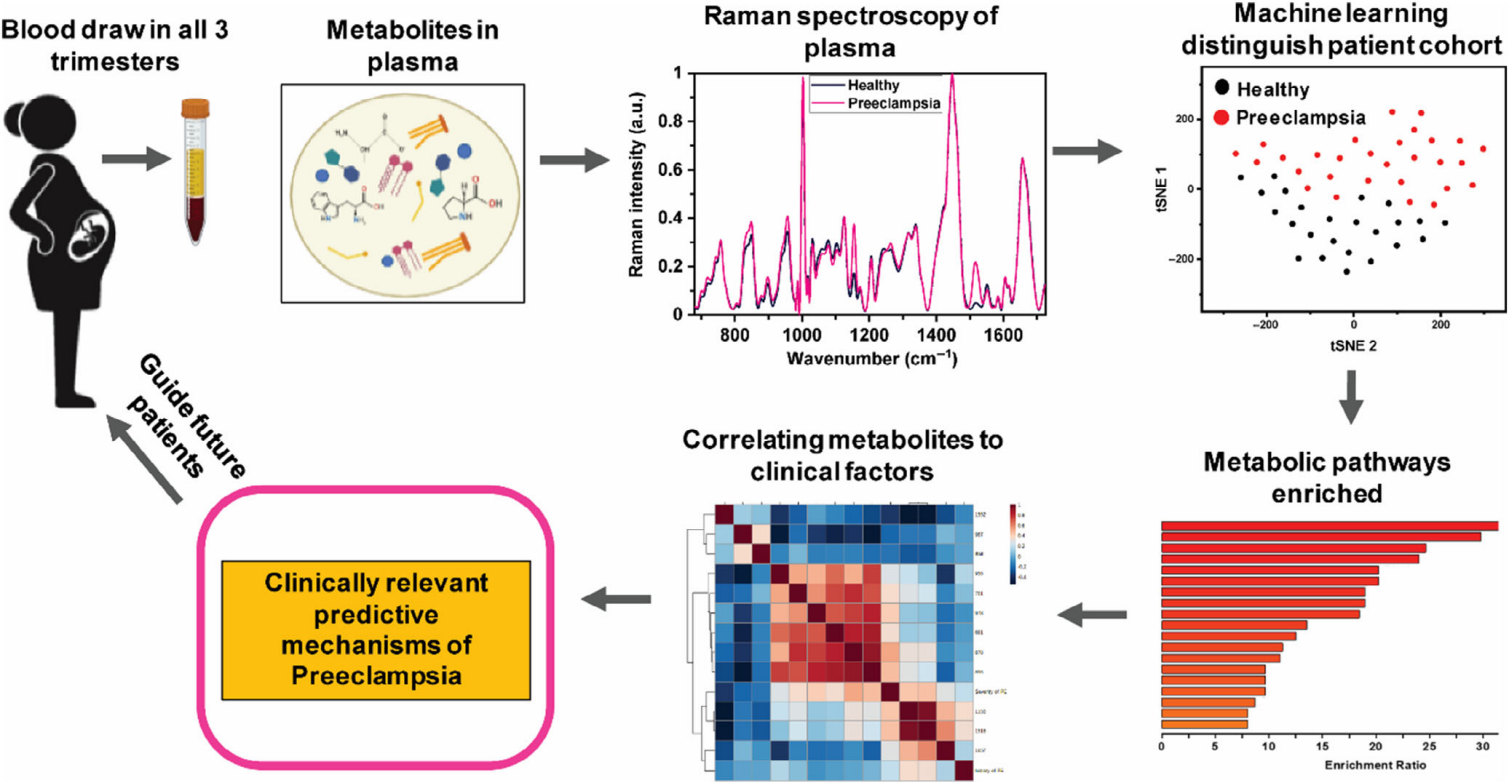
Schematic showing Raman spectra of pregnant patient plasma identify key metabolites that differentiate healthy and preeclampsia patients and identify metabolic pathways enriched. Metabolites are correlated to patient clinical information to understand predictive mechanisms of preeclampsia as early as 1st trimester and guide future patients on therapeutic choices.

In this work we use Raman spectroscopy to assess the metabolic changes in the plasma of pregnant patients in all three trimesters and compare healthy pregnancies to those who were diagnosed with preeclampsia. Unsupervised machine learning, t‐distributed stochastic neighbor embedding (tSNE), integrated with Raman data distinguished the two patient cohorts with >82% accuracy as early as first trimester; the accuracy improved in later trimesters. The advantages of using tSNE lie in its ability to capture complex nonlinear relationships and reveal intricate patterns in high‐dimensional data. Unlike PCA, which primarily focuses on capturing global linear variations, tSNE can detect global and local data structures. This is particularly useful when dealing with complex datasets, as it can uncover subtle differences between the cohorts that may not be apparent with PCA alone. By preserving local neighborhood information, tSNE can highlight clusters or subgroups within each cohort, providing a more granular understanding of the data distribution. In conclusion, tSNE is suitable for Raman data because it can process high‐dimensional data and other Raman papers have also used this method.^31^, ^32^ We identified key metabolites that were altered through pregnancy and using the Kyoto Encyclopedia of Genes and Genomes (KEGG) pathway analysis we determined the corresponding metabolic pathways that show significant differences in preeclamptic patients relative to healthy. We found multiple metabolites have moderate to strong correlations to the patients' severity of preeclampsia, and to other clinical and obstetric factors in all three trimesters. We show that analysis of standard proinflammatory cytokines is not predictive of the onset of preeclampsia, and the sFlt‐1/PlGF ratio is inadequate in ruling out the possibility of preeclampsia in early pregnancy but is accurate in the 3rd trimester. Our work demonstrates that Raman metabolic profiling is a powerful approach in early prediction of preeclampsia and prognosis of the severity of the disease. We envision that in the future, Raman spectroscopy could be integrated in the clinic complimenting current clinical tests to enable early intervention and preventive measures for at risk preeclampsia patients (Scheme 1).

## 2 RESULTS AND DISCUSSIONS

In this study we evaluated plasma samples from pregnant patients throughout the three trimesters who had normal pregnancy (healthy) and those who were diagnosed with preeclampsia. The coded samples were received from Perinatal Family Tissue Bank from the University of Iowa, and we measured a total of 45 samples in the 1st trimester, 45 in the 2nd trimester, and 53 in the 3rd trimester. Complete set of samples for each patient, that is for all three trimesters was not always available from the bank that resulted in variations in sample number among trimesters. Table 1 summarizes the average maternal demographic data including maternal age, body mass index (BMI), number of pregnancies (gravida), number of births (parity), number of pregnancy losses, gestational age at delivery, chronic hypertension, current hypertension, history of preeclampsia, and severity of preeclampsia for both healthy and preeclamptic patients. This information for individual coded patients in the two cohorts is provided in the supporting information (Tables S1 and S2).

**TABLE 1:** Demographics and clinical information of patients.

Clinical parameter	Healthy (mean ± SD)	Preeclampsia (mean ± SD)	*p*‐value
Maternal age	31.2 ± 4.5	32.0 ± 5.9	0.324
BMI	30.8 ± 11.7	28.3 ± 6.9	0.247
Gravida	2.7 ± 1.7	1.9 ± 1.5	0.237
Parity	1.2 ± 0.9	0.7 ± 1.2	0.222
Pregnancy loss	0.5 ± 0.5	0.3 ± 0.94	0.565
Gestational age at delivery (weeks)	38.47 ± 1.95	36.23 ± 2.62	0.0001
Chronic hypertension	11%	20%	0.557
Current hypertension	11%	10%	0.810
History of PE	3%	22%	0.013
Severity of PE	–	57% severe; 42% mild	–

*Note*: Chronic hypertension, current hypertension and the history of preeclampsia are shown as the percentage of patients that had those characteristics.

Abbreviation: BMI, body mass index.

**FIGURE 1:**
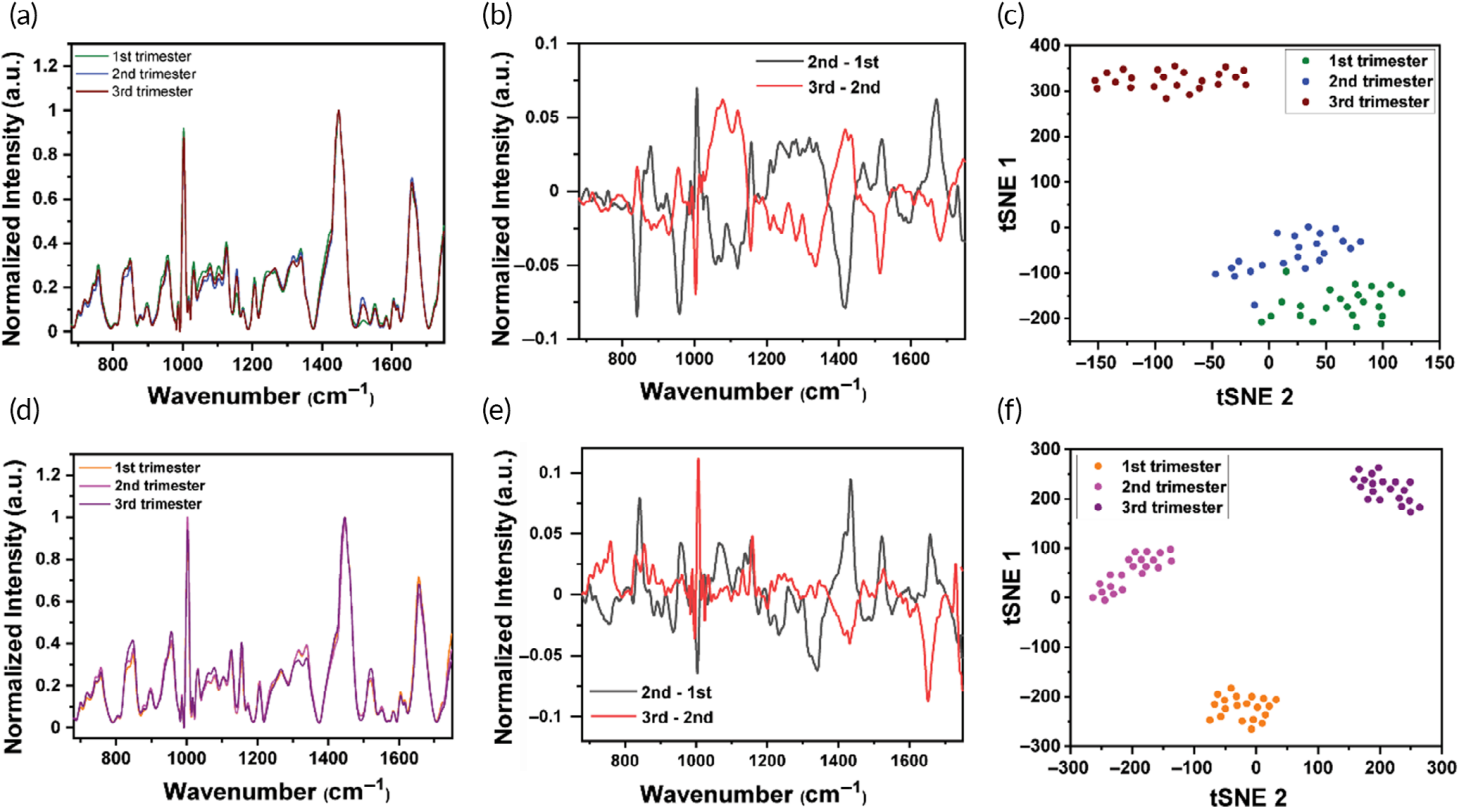
Raman spectroscopy tracks metabolic changes throughout pregnancy in patient plasma. (a) Representative spectra of healthy patient throughout three trimesters. (b) Difference spectrum is obtained by subtracting the spectrum of the 1st trimester healthy from the 2nd trimester (black), and 2nd trimester healthy from the 3rd trimester (red). (c) T‐distributed stochastic neighbor embedding (tSNE) of all healthy samples shows separation between trimesters. Healthy 1st trimester *n* = 23, healthy 2nd trimester *n* = 25, healthy 3rd trimester *n* = 24. Each dot represents the averaged spectrum of one patient. (d) Representative spectra of preeclampsia patient throughout three trimesters. (e) Difference spectrum of preeclampsia patient similar to that shown for healthy in “b.” (f) tSNE of all preeclampsia samples shows separation between trimesters. Preeclampsia 1st trimester *n* = 22, preeclampsia 2nd trimester *n* = 20, preeclampsia 3rd trimester *n* = 29.

The Raman spectra of the healthy and preeclampsia samples were measured by a 120 mW, 785 nm laser under a 50× objective. Three microliters of the thawed plasma was aliquoted on a CaF_2_ disk and dried for 20 min in 37°C and immediately measured. The raw spectra were processed and normalized with the standard normal variate (SNV) method in a custom MATLAB code, and again renormalized so that the 1445 cm^−1^ peak would have a value of 1 across all samples. We chose this peak for normalization as it had minimal variation across samples. We first aimed to show Raman is effective in tracking metabolic changes throughout pregnancy in both patient cohorts. The normalized Raman spectra of samples (Figure 1a,d; Figures S1–S3 supporting information) show longitudinal changes in plasma metabolites throughout the three trimesters, which is captured in the difference spectrum (Figure 1b,e). The two difference spectra were obtained by subtracting the 1st trimester spectra from the 2nd trimester, and by subtracting the 2nd trimester spectra from the 3rd trimester, respectively. Positive values in the difference spectra represent an increase in the abundance of a specific metabolite and negative values represent a decrease in those metabolites in the trimesters represented. The Raman peaks, the tentative metabolic assignment, and the corresponding vibrational modes are listed in Table 2 confirmed with literature findings.^33^, ^34^, ^35^, ^36^, ^37^ In assigning our peaks, we used a three step approach: (1) we referred to the cited literature in Table 2, which are highly cited and well‐established Raman reference papers^33^, ^34^, ^35^, ^36^, ^37^; (2) we referred to papers focused on metabolism in pregnancy disorders (see references 38, 39, 40, 41, 42, 43, 44, 45, 46, 47, 48, 49, 50, 51, 52, 53) to ensure that the metabolites assigned are relevant in pregnancy; and (3) we also referred to papers that highlight metabolites present in blood and blood components.^54^, ^55^ This three step approach gave us confidence in our tentative peak assignments, and the metabolic pathways that have resulted from these assignments. For metabolites with multiple peak assignments (such as many of the amino acids that have multiple peaks), we only considered the strongest Raman peak of that metabolite as weaker peaks are unlikely to appear in a mixed media such as plasma.

**TABLE 2:** Peak assignments and corresponding vibrational modes of Raman spectral data.

Wavenumber (cm^−1^)	Tentative peak assignment	Vibrational modes	Ref.
681	Methionine	CO_2_‐scissoring and deformation	33, 34
701	Cholesterol	Choline group, CH_2_ rocking	35, 36
718	Phosphatidylcholine	Asymmetric stretching of N+(CH_3_)_3_	35, 37
743	Thymine (DNA bases)	Backbone vibrations, deformation of the ring	34, 35
757	Tryptophan	Symmetric breathing of tryptophan, σ(ring)	33, 35
805	DNA, RNA	Symmetric breathing, C—C stretching	34, 35
835	Tyrosine	Asymmetric O—P—O stretching	33, 35
848	Sugars (Glucose, Glycerol)	C—O—C skeletal stretching	34, 35
876	Glutamic acid	C—O—C ring, C—O—H bending	33, 34
898	Glycine	CH_2_ wagging vibrations from backbone	33, 34
940	Citric acid, Succinic acid	υ(OH…O) out of plane wagging vibration of intermolecular hydrogen bonds	34
956	Lipids	C—H bending, C—C, and C—O stretching	36
987	Arginine	C—N stretching	33, 34
1002	Phenylalanine	Symmetric ring breathing	35
1017	Carbohydrates	C—O—C ring, C—O—H bending	35
1031	Phenylalanine	C—H in‐plane bending	33, 35
1056	Lipids	Chain C—C stretching	35, 36
1078	Lipids	Chain C—C stretching	35, 36
1105	Mannose, Trehalose	σ(CH_2_) twisting vibrations	35
1126	Glucose	C—N stretching	34, 35
1154	Carotenoids	C—C and C—N stretching	35, 37
1171	Saturated long‐chain fatty acids	C—C stretching (skeletal option)	35, 36
1206	Amino acids	NH_3_ asymmetric rocking	33, 34
1243	Amide III	Asymmetric phosphate stretching modes	35, 37
1265	Unsaturated lipids and fatty acids	C—C and C—H stretching	35, 36
1303	Triglycerides	CH_3_ CH_2_ twisting	33, 34
1317	Histidine	C—S stretching, CH_3_/CH_2_ twisting or bending mode	33, 34
1340	Threonine	CH_3_/CH_2_ twisting/wagging mode	33
1355	Isoleucine	CH_3_/CH_2_ wagging	33
1420	DNA	C—S stretching of cytosine, C—C stretching, ring breathing mode	34, 35
1517	Carotenoids	C—C and C—N stretching	35, 37
1551	Tryptophan	Symmetric breathing, C—C stretching	33, 34
1584	Phenylalanine	C—C bending	33, 35
1606	Phenylalanine	Ring C—C stretching, ring vibration	33, 35
1617	Tyrosine	C=C stretching mode of tyrosine	33, 34, 35
1657	Unsaturated lipids, phosphatidylcholine, phosphatidylethanolamine	C—C and C—O stretching	35, 36, 37
1672	Amide I	C—C and C—O stretching	35

Interestingly, the difference spectra show that the intensities of many Raman peaks change in an opposing manner across the three trimesters in both the healthy and preeclampsia cohorts that we will discuss in details in Figure 3. We performed a student *t*‐test on the Raman peak values to determine which metabolites were significant across the three trimesters for both patient cohorts and *p*‐value <0.05 were considered statistically significant. We then applied tSNE, a nonlinear dimensionality reduction approach, that with proper choice of hyperparameters can also enable data clustering and separation based on similarity and dissimilarity respectively, in the original higher‐dimensional space (Figure 1c,f).^56^ In the tSNE plots each point represents a single patient's averaged and normalized spectrum, and an exaggeration of 3 was used to separate the data into the three trimesters. The clear separation observed in tSNE plots for both healthy and preeclampsia cohorts suggests that: (i) metabolic processes during pregnancy are both dynamic and precisely programmed, and these changes can be successfully captured by RS throughout the three trimesters. (ii) In the healthy cohort, a pronounced separation of the 3rd trimester metabolic data relative to 1st and 2nd is observed, but in preeclampsia cohort all three trimesters are clustered separately. This emphasizes that an increase in fetal development in the 3rd trimester alters the maternal metabolome later in pregnancy significantly. However, dysregulated metabolic processes in preeclampsia likely contributes to the distinct clustering of data in tSNE for all three trimesters. Therefore, next we aimed to compare the metabolic changes between healthy and preeclampsia in each trimester and identify the key metabolites that are early indicators of the onset of preeclampsia.

**FIGURE 2:**
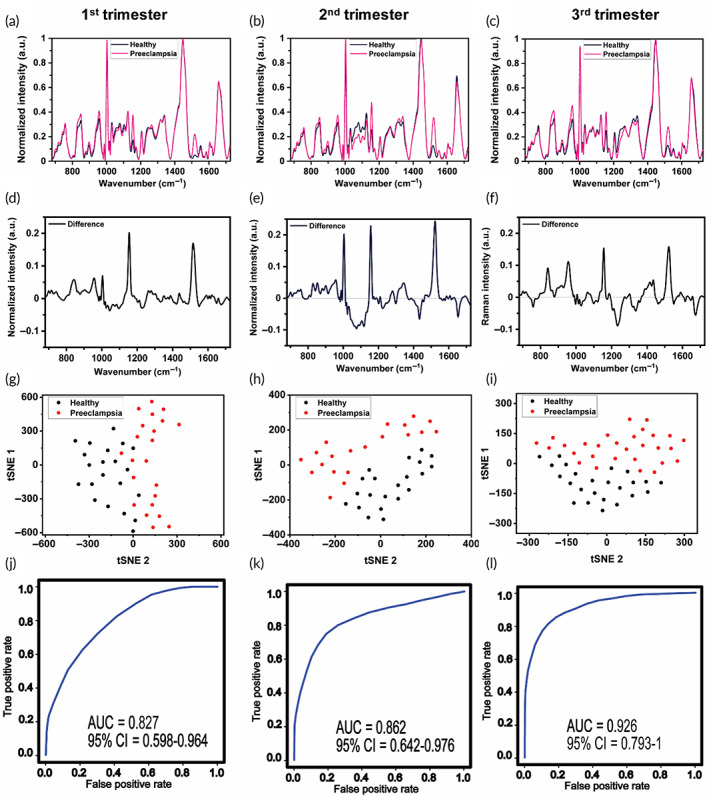
Raman spectroscopy shows the differences in metabolites' in healthy (black) versus preeclampsia (pink) patients in each trimester. (a–c) Mean, normalized Raman spectra of a healthy and a preeclampsia patient normalized to the 1447 cm^−1^ lipid peak for the 1st, 2nd, and 3rd trimester from left to right. (d–f) The difference spectrum is obtained by subtracting the healthy spectrum from the preeclamptic spectrum in “a–c.” (g–i) tSNE of all the healthy (black) and preeclampsia (red) samples where each dot represents the averaged data of one patient. A perplexity of 11, 10, and 18 with 10, 6, and 5 PCA components were used for the 1st, 2nd, and 3rd trimester, respectively. (j–l) An AUC‐ROC curve obtained with a weighted SVM model shows the predictability rates of 0.827, 0.862 and 0.926 for 1st, 2nd, and 3rd trimester, respectively.

The Raman spectra of healthy and preeclampsia cohorts (Figures 2a–c and S1–S3) and the corresponding difference spectra (Figure 2d–f), obtained by subtracting the healthy spectrum from the preeclamptic spectrum, show the dynamic profile of metabolic changes longitudinally across the three trimesters. By performing a student *t*‐test on the Raman peak values, we determined 12, 15 and 17 statistically significant metabolites in the 1st, 2nd and 3rd trimester respectively, these peaks can be seen in Table S10. The significant peaks were used in tSNE analysis (Figure 2g–i) that shows healthy and preeclampsia patient samples were clearly clustered in all three trimesters with the strongest separation in the 3rd trimester. In tSNE each point represents a single patient's averaged and normalized spectrum. To ensure that the separation observed in tSNE is an accurate classification of the Raman data, we generated an area under the curve–receiver operating characteristic curve (AUC‐ROC) for each trimester (Figure 2j–l). The ROC analysis verifies the correct classification of data based on the sensitivity and specificity of the corresponding Raman peaks. The sensitivity is the true positive rate, or the percentage of patients with preeclampsia. The specificity is the true negative rate or the percentage of healthy patients. A weighted support vector machine (SVM) algorithm was used to generate the AUC‐ROC curves using MetaboAnalyst, a free online tool for metabolic profiling.^57^ The Raman data achieved an AUC of 0.827 with a 95% confidence interval (CI) of 0.598–0.964 in the 1st trimester, an AUC of 0.862 with a 95% CI of 0.642–0.976, and an AUC of 0.926 with a 95% CI of 0.793–1 in the 3rd trimester. Our AUC results demonstrate that RS can determine which patients are likely to develop preeclampsia with high accuracy as early as first trimester, which is not possible through existing clinical tests.

**FIGURE 3:**
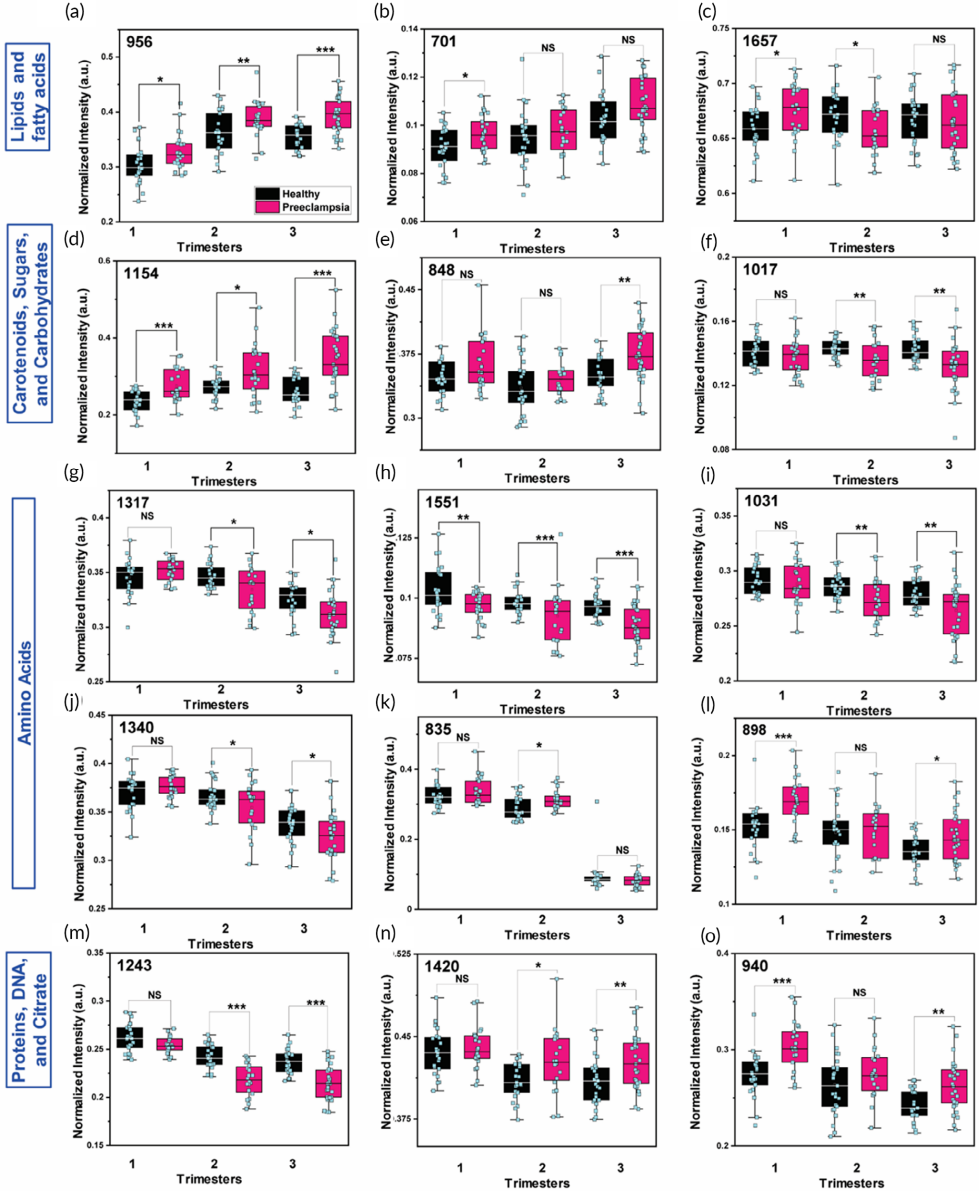
The most relevant metabolites in all three trimesters are presented in box plots based on their metabolic class for healthy (black) and preeclampsia (pink) patient cohort. (a–c) Lipids and fatty acids: lipids/myristic acid (956 cm^−1^), cholesterol (701 cm^−1^), phospholipids/unsaturated lipids (1657 cm^−1^). (d–f) Carotenoids, sugars, and carbohydrates: β‐carotene (1154 cm^−1^), sugars (848 cm^−1^), carbohydrates (1017 cm^−1^). (g–l) Amino acids: histidine (1317 cm^−1^), tryptophan (1551 cm^−1^), phenylalanine (1031 cm^−1^), threonine (1340 cm^−1^), tyrosine (835 cm^−1^), and glycine (898 cm^−1^). (m–o) Proteins: Amide (III) (1243 cm^−1^), DNA (1420 cm^−1^), and citric/succinic acid (940 cm^−1^). Statistical significance between each cohort is noted with stars. Here, * represents a *p* value <0.05, ** represents a *p* value <0.01 and, *** represents a *p* value <0.001 and NS stands for not significant.

Next, we examined the trends in the most important metabolites across the three trimesters in the two patient cohorts (Figures 3 and S4) based on the statistically significant peaks of RS. We determined 12, 15 and 17 significant peaks in 1st, 2nd and 3rd trimester, respectively. We observed alterations in multiple classes of metabolites: lipids and fatty acids (FAs); carotenoids, carbohydrates and glucose; amino acids (AAs); tricarboxylic acid (TCA) cycle; DNA; and proteins. Within the first class, the increase in lipid peak at 956 cm^−1^ (Figure 3a) in preeclampsia samples was expected since increased plasma lipid levels are known to increase the risk of cardiovascular diseases and hypertension through endothelial cell dysfunction.^38^ Literature evidence show an increase in most lipids except low‐density lipoproteins.^38^, ^39^ Cholesterol (Figure 3b) generally shows an increasing trend in all three trimesters but most significant early in pregnancy. Whereas cholesterol is essential to fetal growth, high levels of cholesterol have been correlated to increased oxidative stress and inflammation resulting in endothelial cell dysfunction and lesions in placental vasculature that are characteristic of preeclampsia pathophysiology.^40^ The peak at 1657 cm^−1^ (Figure 3c) assigned to unsaturated lipids and phospholipids including phosphatidylcholine/phosphatidylethanolamine (PC/PE) shows an ambiguous trend with an increase in preeclampsia in the 1st trimester but a decrease in the 2nd trimester. This may suggest an increase in fetal consumption of FAs and phospholipids likely depletes them in maternal circulation as free FAs and lipids are known to readily cross the placenta, and the changes are more drastic in preeclamptic cohort.^41^ It is important to note that there are many lipids and FAs in the maternal metabolome with variable changes during pregnancy reflecting their complex crosstalk with the signaling molecules secreted from the placenta.

Within the next class of metabolites, we observe an increase in β‐carotene (1154 cm^−1^, Figure 3d) in preeclampsia samples throughout pregnancy. Carotenoids are naturally found in fruits and vegetables and many prenatal vitamins. The increase in β‐carotene could be correlated to the diet or intake of supplements by patients. Since carotenoids are antioxidants, we would expect β‐carotene would alleviate the overly oxidized or inflamed state of preeclampsia and therefore, decrease in intensity.^42^ However, the opposing trend in our results suggests an alternative hypothesis. Carotenoids serve as the precursor for synthesis of vitamin A required for the cellular growth of the developing fetus. Since vitamin A is not synthesized de novo by the embryo, it relies on circulating maternal vitamin A that crosses the placenta to reach the fetus. Placental insufficiency, which is prevalent in preeclampsia, obstructs successful transfer of maternal nutrients including vitamin A to the fetus.^43^, ^44^ Disruption of this maternal–fetal homeostasis may manifest as accumulation of β‐carotene in the maternal plasma which explains the increase in carotenoids in preeclampsia samples. We observed a similar trend in carotenoids in preterm labor pregnancies relative to healthy suggesting the important role of β‐carotene during pregnancy.^28^ We also observed that sugar levels (848 cm^−1^, Figure 3e) are higher in preeclampsia cohort in all three trimesters relative to healthy. Indeed, there is a strong relationship between abnormal glucose metabolism and hypertension during pregnancy, and preeclamptic patients often have higher blood glucose levels that are exaggerated closer to term.^45^ Further, preeclampsia and gestational hypertension have also been identified as a risk factor for gestational diabetes.^46^ An increase in sugars correlate well to the decrease in carbohydrates peak at 1017 cm^−1^ (Figure 3f) as carbohydrates are a key player in the energy metabolism and serve as a precursor for glucose synthesis.^47^

Within the class of AAs, we observe a number of essential and nonessential AAs (Figure 3g–j) decrease in the preeclampsia cohort including histidine (1317 cm^−1^), tryptophan (1551 cm^−1^), phenylalanine (1031 cm^−1^), and threonine (1340 cm^−1^). AAs are important building blocks in the protein synthesis chain and are crucial for fetal growth and placental function. Maternal AAs are actively transported across the placenta for fetal growth; as a patient nears term, a significant increase in fetal AA consumption likely depletes certain AAs from maternal circulation as observed in our results.^48^ A decrease in some AAs in preeclamptic patients suggest a decreased expression of placental AA transporters, dysregulated maternal–fetal tolerance, and placental insufficiency which are all hallmarks of preeclampsia. Therefore, a decrease in these AAs is an early indicator of cervical changes that also leads to preterm labor as noted for many the preeclampsia patients (Table 1). For example, histidine is a precursor of the antioxidant carnosine, so a decrease in histidine in preeclampsia in the 2nd and 3rd trimester may suggest conversion of histidine to carnosine to compensate for excessive oxidative stress found in preeclampsia.^49^ But we also observe some AAs increase in one or more trimesters (Figure 3k,l) including tyrosine (835 cm^−1^), and glycine (898 cm^−1^). An increase in glycine in preeclamptic patients has been associated with glycolysis defects in the placenta and correlates well with the increase in glucose observed in our results (Figure 3e).^50^ This suggests that AAs have a complex interplay in pregnancy both necessary for fetal growth but also contribute to inflammation and dysregulation of energy pathway. In our previous work focused on Raman spectral analysis of pregnant patient plasma samples who had preterm birth, we also observed similar tends in AAs.^28^ Metabolic rewiring in AAs also contributes to disrupted protein synthesis and corroborates with the statistically significant decrease we observe in amide III peak at 1243 cm^−1^ (Figure 3m) that represents proteins and type I collagen. Indeed literature evidence shows that in preeclampsia multiple closely‐packed proteomic pathways within the protein networks and corresponding gene expression are simultaneously dysregulated.^51^ These disrupted pathways may contribute to placental hypoxia, endothelial dysfunction, and even premature cervical ripening and parturition. We also observe an increase in peaks corresponding to DNA (1420 cm^−1^, Figure 3n) in the 2nd and 3rd trimesters in preeclamptic patients that may correspond to an elevation of cell‐free fetal DNA in maternal circulation.^52^, ^53^ Abnormalities in placental development leads to apoptosis and alterations in placental cell composition releasing fetal DNA from the placenta into maternal circulation and due to impaired liver and kidney function in this inflammatory disease, cell‐free DNA is cleared slowly from the bloodstream.^58^ We observe that citric/succinic acid, intermediate metabolites in the TCA cycle, (940 cm^−1^, Figure 3o) has a decreasing trend through pregnancy, but in each trimester it is amplified in the preeclamptic cohort. An increase in citric acid, which is associated with oxidative stress, aligns well with the hypoxic state of preeclampsia.^59^

**FIGURE 4:**
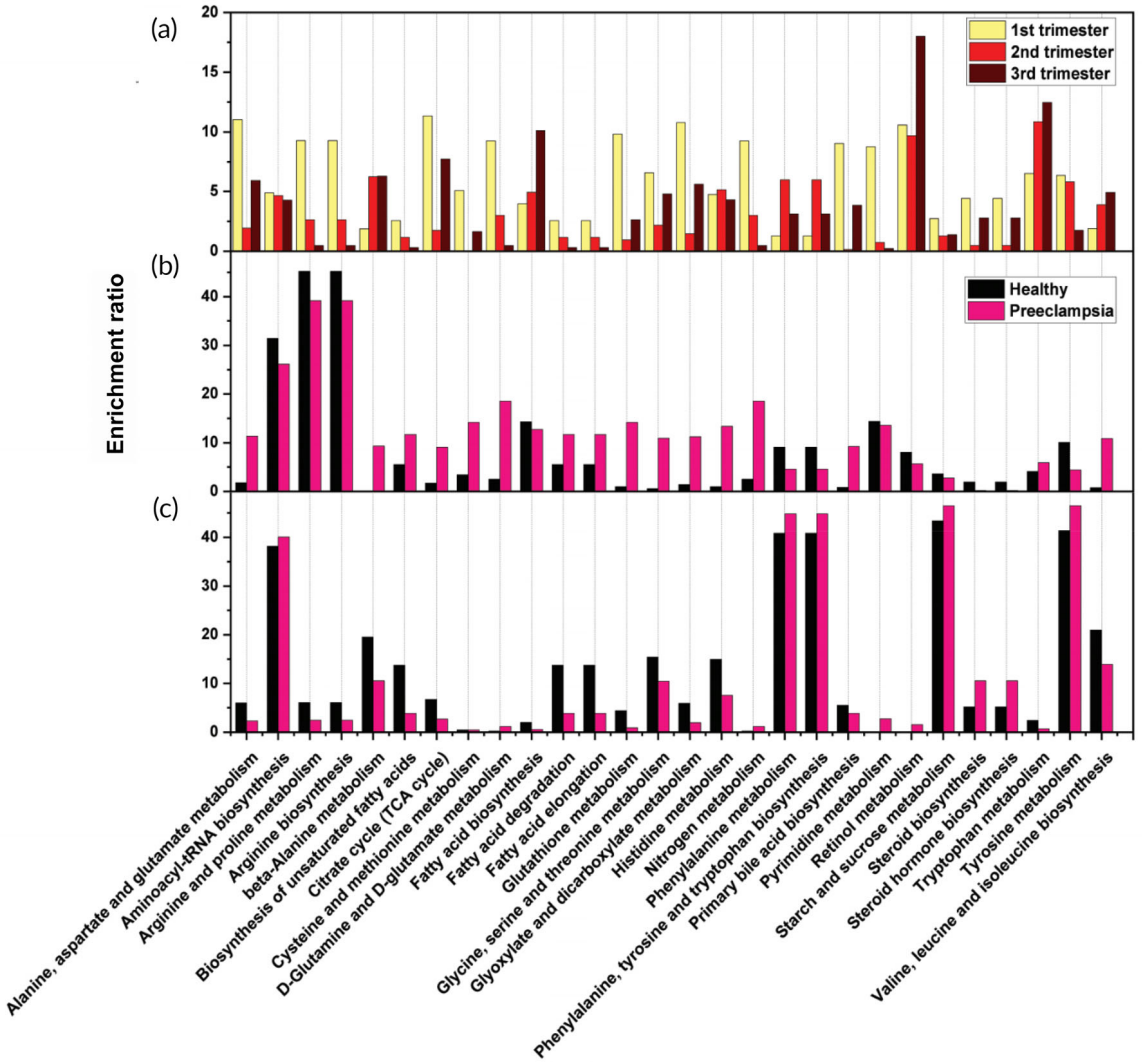
KEGG pathway enrichment analysis of complete sample cohorts (1st trimester *n* = 45, 2nd trimester *n* = 45, 3rd trimester *n* = 53) showing (a) Enrichment analysis through the 1st, 2nd and 3rd trimester of pregnancy. (b) Enrichment ratios of the most important metabolic pathways changing from the 1st trimester to the 2nd trimester in preeclampsia (pink) versus healthy (black). (c) Enrichment ratios of the most important metabolic pathways changing from the 2nd trimester to the 3rd trimester in preeclampsia (pink) versus healthy (black). For “b” and “c” we used data from *n* = 23 healthy patients and *n* = 22 preeclampsia patients from 1st trimester, *n* = 25 healthy and *n* = 20 preeclampsia patients from 2nd trimester, and *n* = 24 healthy and *n* = 29 preeclampsia patients from 3rd trimester.

The metabolites identified with RS were used to map out the corresponding metabolic pathways via KEGG pathway analysis that are enriched in both healthy pregnancy and in preeclampsia throughout pregnancy (Figure 4). The specific metabolites involved in each of these enrichment pathways can be found in Table S9 in the supporting information. In Figure 4a we show pathways that are enriched in all of the patients in both healthy and preeclampsia to see the trends across pregnancy. We find some metabolic pathways associated with AAs show an increasing trend from 1st to 3rd trimester including metabolism of β‐alanine, phenylalanine, tryptophan, and valine/leucine/isoleucine. While other AA pathways show a decreasing trend as the pregnancy progresses that include metabolism of alanine, aspartate, and glutamate, arginine and proline, arginine biosynthesis, cysteine and methionine, and glutamine. For example, L‐arginine is a key contributor of oxidative stress by increasing reactive nitrogen species; further, endothelial dysfunction in preeclampsia has been correlated to aberrant arginine metabolism.^60^, ^61^, ^62^ These opposing trends highlight the complex role of AAs discussed previously. Pathways associated with citric acid (TCA cycle; glyoxylate and dicarboxylate metabolism) also show a decreasing trend across pregnancy as observed in our Raman analysis (Figure 3o). These pathways are the main source of cellular energy; as the pregnancy advances, fetal and placental growth have a high energy demand resulting in depletion of maternal citric acid from 1st to 3rd trimester.^63^

Next, we examine which metabolic pathways are rewired in preeclampsia relative to healthy as patients' transition from 1st to 2nd trimester (Figure 4b), and then from 2nd to 3rd trimester (Figure 4c). Some enrichment ratios from the 1st to the 2nd trimester show a drastic increase in the preeclamptic cohort including multiple metabolic pathways associated with AAs such as glutamate, TCA cycle, fatty acid degradation, and nitrogen metabolism among others. Glutamate, which originates from the placenta, is among the most utilized AAs in fetal growth. Glutamate is cleared from fetal plasma and taken up by the placenta where it is converted to glutamine and then transferred back into fetal circulation.^64^ Glutamate synthesis is associated with nitrogen metabolism where branched chain amino acids such as leucine/isoleucine serve as nitrogen pool. This suggests these metabolic pathways are closely intertwined and an enrichment of these pathways in maternal plasma in preeclampsia suggests dysregulated transport of nutrients across the fetal–placental–maternal interface. We also observe pathways corresponding to retinol metabolism decrease in preeclampsia. Retinol or Vitamin A plays a vital role in growth and development of embryonic cells and deficiencies of this metabolite has been associated with fetal growth restriction, which is strongly associated with severity of preeclampsia.^65^, ^66^ We observe an increase in FA degradation and FA elongation in preeclampsia. FAs have multiple role in preeclampsia; synthesis of unsaturated FAs such as omega 3 and omega 6 are essential for placental function and for regulating oxidative stress and inflammation.^67^ Further, the placenta derives energy from mitochondrial fatty acid oxidation (FAO) that controls both maturation of the placenta, and fetal growth. Defects in FAO pathway at the maternal–fetal interface has been correlated to accumulation of toxic metabolic precursors that are transferred to the maternal circulation and has been associated with preeclampsia.^68^ The bile acid synthesis pathway also increased in the preeclamptic cohort supported by literature findings that correlates hypertensive disorders of pregnancy to liver function.^69^ Bile acid accumulation in maternal circulation negatively impacts the expression of placental angiogenic and antiangiogenic factors that alters vascular remodeling, elevates proinflammatory cytokines and chemokines in preeclampsia. These findings indicate that these pathways reprogrammed in preeclampsia may serve as early markers to predict onset of preeclampsia.

As patients progress from 2nd to 3rd trimester, several interesting trends in metabolic pathways are observed. We find that some AA metabolic pathways including tyrosine, phenylalanine, and phenylalanine/tyrosine/tryptophan biosynthesis are enriched later in pregnancy in both cohorts suggesting that an increase in fetal consumption of AAs encourages higher production of AAs by the mother. But in preeclampsia, aberration in these pathways leads to poor transport of AAs to the fetus and therefore accumulation in maternal circulation. We also find some AA metabolism that were enriched earlier in pregnancy are less relevant later in pregnancy (arginine biosynthesis, arginine and proline, cysteine and methionine, glutamine) suggesting these pathways are primarily needed for early placental development. Starch and sucrose metabolism is also highly enriched later in pregnancy which is necessary for the growing fetus. This pathway is also higher in preeclampsia which correlates with the increase in glucose observed in our Raman results (Figure 3e). Literature also suggests high levels of circulating aromatic AAs (phenylalanine/tyrosine/tryptophan biosynthesis) is also associated with high plasma glucose and the risk of diabetes that is increased in preeclampsia.^70^ We also observe a decrease in biosynthesis of unsaturated fatty acids in the preeclamptic cohort, which corresponds well with literature findings, and may play a role in the pathogenesis of preeclampsia.^71^ Finally, we observe an elevation in steroid metabolic pathways in preeclampsia; studies show increased testosterone and progesterone but decreased estradiol in the preeclamptic serum.^72^ Progesterone increases the production of endothelial vasodilators, such as nitric oxide, which is detrimental to vascular smooth muscle cell growth and placental stability as observed in preeclampsia.^73^

**FIGURE 5:**
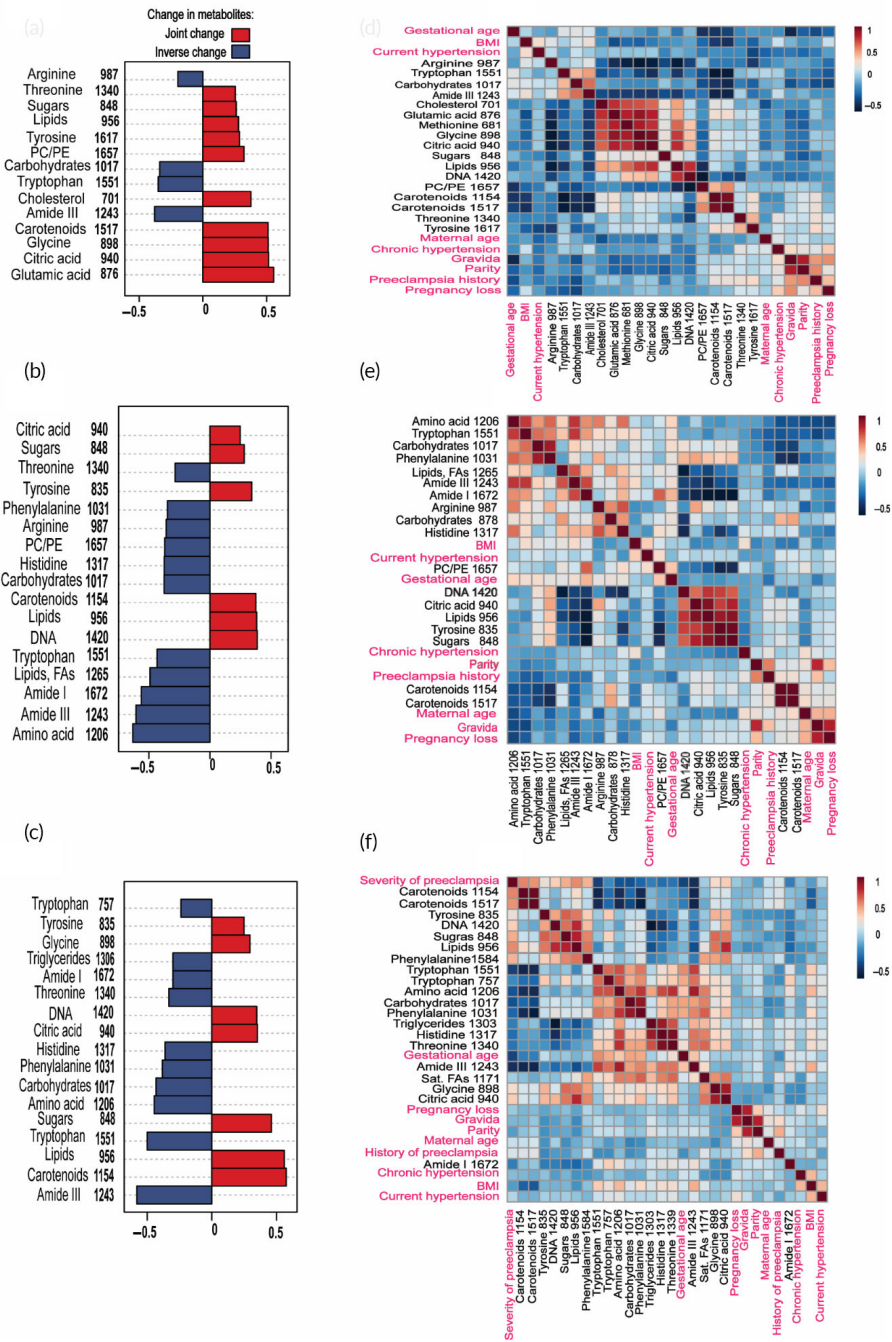
Pearson's correlation plots and heatmaps correlating Raman data with clinical data in every trimester using total number of samples in every cohort (1st trimester *n* = 45, 2nd trimester *n* = 45, and 3rd trimester *n* = 53). From top to bottom: 1st, 2nd, and 3rd trimester. (a–c) Statistically significant peaks of Raman in every trimester were correlated to the severity of preeclampsia. Positive values are plotted in red and show a metabolite jointly changing with the severity of preeclampsia, while negative values are plotted in blue and show metabolites inversely changing with the severity of preeclampsia. (d–f) Correlation heatmaps showing the statistically significant Raman peaks in the (d) 1st, (e) 2nd, and (f) 3rd trimester correlated to patient clinical and obstetric information including maternal age, BMI, gravida, parity, gestational age at delivery, history of preeclampsia, chronic and current hypertension, and pregnancy loss. FA, fatty acid; PC/PE, phosphatidylcholine/phosphatidylethanolamine; sat, saturated; TG, triglycerides.

We then performed correlation analysis to understand the relationship of the different metabolites identified with RS and the severity of preeclampsia. To quantify clinical data, values of 0, 1, 2 and 3 were assigned respectively, to healthy samples, low preeclampsia, mild preeclampsia, and severe preeclampsia (including the HELLP syndrome and superimposed preeclampsia). All these values were then normalized between 0 and 1 to match the range of our normalized Raman data. These normalized values were then used to determine a Pearson product–moment coefficient (*r*) as a measure of correlation (Figure 5a–c **)** where positive values suggest that those peaks change jointly with the severity of preeclampsia and negative values show an inverse change with respect to the severity of preeclampsia. These correlation ratios for every metabolite can be found in the supporting information Tables S3–S5. Values between ± (0–0.29) suggest a weak correlation, ± (0.30–0.50) moderate correlation, and ± (0.51–1) strong correlation of variates to each other. These ranges for correlation strengths were adapted from the literature.^74^, ^75^ Our results show that in the first trimester (Figure 5a) glutamic acid (*r* = 0.555), citric acid (*r* = 0.516), glycine (*r* = 0.512), and carotenoids (*r* = 0.510) have strong joint correlation to the severity of preeclampsia, whereas amide III (*r* = −0.378), tryptophan (*r* = −0.350), and carbohydrates (*r* = −0.338) have moderate inverse correlation to severity of preeclampsia. In the 2nd and 3rd trimester majority of the metabolites and proteins represented in Figure 5b,c have moderate to strong correlation to severity of preeclampsia. These findings indicate that longitudinal metabolic monitoring throughout pregnancy is key to early prediction of patients who may develop preeclampsia and may provide guidance on monitoring these patients.

We then examined if metabolites measured throughout pregnancy correlated with patients' clinical information that include maternal age, BMI, gravida, parity, gestational age at delivery, history of preeclampsia, chronic and current hypertension, and pregnancy loss (Figure 5d–f). The correlation ratios for each of these heatmaps in Figure 5d–f can be found in the supporting information, Tables S6–S8. Normalized values for Raman and clinical information were used similar to Figure 5a–c. We generated a correlation heat map where blue cells represent an inverse correlation and red cells represent joint correlation. In the 1st and 2nd trimester heatmaps we excluded severity of preeclampsia in current pregnancy in order for the analysis to apply to patients who have not yet been diagnosed with preeclampsia. However, most preeclamptic patients are clinically diagnosed by the 3rd trimester, therefore severity of preeclampsia in current pregnancy was included in the 3rd trimester heatmaps. Our findings show that in the 1st trimester (Figure 5d), an inverse correlation exists between BMI and both carotenoid peaks (1155, 1517 cm^−1^). Indeed clinical studies have shown an inverse relationship of carotenoids and BMI where the anti‐obesity effect of β‐carotene is linked to the regulation of adipose tissue through the PPAR‐γ (peroxisome proliferator‐activated receptor γ) pathway; adipose tissue is a key player in obesity and the main storage tissue for carotenoids.^76^, ^77^ We also find an inverse correlation of carotenoids to gestational age at delivery, and joint correlation to the history of preeclampsia emphasizing the importance of β‐carotene to maintain healthy pregnancy. BMI also has a positive correlation to current hypertension, which is expected as individuals with high BMI are at a higher risk of developing hypertension.^78^

In the 2nd trimester (Figure 5e) BMI continues to be inversely correlated to carotenoids. Further, we find that whereas in the 1st trimester no correlation was found between metabolites and maternal age, gravida, parity, and pregnancy loss, in the 2nd trimester these obstetric factors have moderate to strong correlation to multiple metabolites. Tryptophan (1552 cm^−1^) for example is inversely correlated to maternal age (*r* = −0.41033), gravida (*r* = −0.49591), parity (*r* = −0.32254), pregnancy loss (*r* = −0.43639), and history of preeclampsia (*r* = −0.42998). Other AA peaks in Raman (835 cm^−1^, 1206 cm^−1^) are also inversely correlated to chronic hypertension and history of preeclampsia indicating AAs are essential to maintain healthy placental function, fetal growth, and manage blood pressure. In the Raman spectra, proteins are represented as the amide I (1672 cm^−1^) and amide III peak (1243 cm^−1^). The amide III peak shows inverse correlation to gravida, pregnancy loss, and history of preeclampsia emphasizing that dysregulated protein synthesis is a key risk factor in preeclampsia. We find gestational age is jointly correlated to proteins (amide I, 1672 cm^−1^) indicating protein synthesis is crucial to maintaining healthy placental function. Gestational age is also jointly correlated to phospholipids (1657 cm^−1^) including PC/PE which aligns well with literature in which choline supplementation improved placental nutrient transport and placental vascular development to enable term delivery.^79^, ^80^ We find an inverse correlation of maternal age to many essential AAs including tryptophan, phenylalanine, threonine, and arginine suggesting patients of advanced age may consider supplementing these AAs to ensure normal infant growth. Finally, maternal age is inversely correlated to citric acid, which was expected as genes related to energy metabolism, including TCA cycle metabolites, are downregulated in elderly adults.^81^

As the pregnancy advances to the final trimester, we find significant correlations among the clinical factors and metabolites, and cross correlation between the two (Figure 5f). Severity of preeclampsia has moderate to strong correlation to multiple metabolites as represented in Figure 5c. Severity of preeclampsia is inversely correlated to gestational age, indicating a higher risk for preeclamptic patients to deliver preterm. We find gestational age also has a joint correlation to carbohydrates (1017 cm^−1^), amide III (1243 cm^−1^) and multiple AAs that suggests those who delivered at term had higher metabolites in maternal circulation to meet the needs of the growing fetus. In the 3rd trimester, BMI continues to be inversely correlated to carotenoids. BMI also has a joint correlation to AAs indicating patients with high BMI may have a poor placental transfer of AAs to the fetus resulting in higher AA in maternal circulation. BMI is also jointly correlated to chronic and current hypertension suggesting that overweight/obese patients are at a higher risk of preeclampsia.^82^, ^83^ Other clinical and obstetric factors have weak correlations to metabolites in the 3rd trimester. However, we find strong correlations among metabolites as pregnancy is very metabolically active in the third trimester. We also find that throughout pregnancy metabolites within the same class (e.g., all metabolites that belong to AAs) as well as metabolites among different classes (e.g., lipids and carbohydrates) have moderate to strong correlations to each other. These trends show that as expected the catabolic pathways for these metabolites are linked.

Collectively our findings show that metabolites are key markers to predict poor maternal and neonatal outcomes in patients' throughout pregnancy. Therefore, patients may benefit from metabolic testing in all three trimesters to a priori determine those at high risk of developing preeclampsia. Our results also show that metabolic measure with Raman spectroscopy is highly effective in addressing an unmet clinical need by accurately predicting the onset of preeclampsia (AUC of 0.827) as early as 1st trimester. Previous findings with mass spectrometry metabolomics in 2nd trimester (~15 weeks gestation) has demonstrated that metabolic profiling with a multivariate model is predictive of preeclampsia. The mass spectrometry studies reported similar classes of metabolites, validating our results utilizing Raman.^84^ Correlation of metabolites measured with Raman to clinical/obstetric factors of patients throughout pregnancy suggest that targeted metabolic screening can be performed in high‐risk women with a history of hypertension as early as the 1st trimester. Finally, Raman spectral analysis enables multiple advantages including rapid measurement (30 min per sample), low cost, no sample preparation needed (thaw samples and measure), and minimal sample volumes (3 μL plasma) required. Therefore, Raman metabolic testing could be implemented as an affordable maternal screening and when combined with patients current clinical factors, it may enhance prenatal care for high‐risk patients.

**FIGURE 6:**
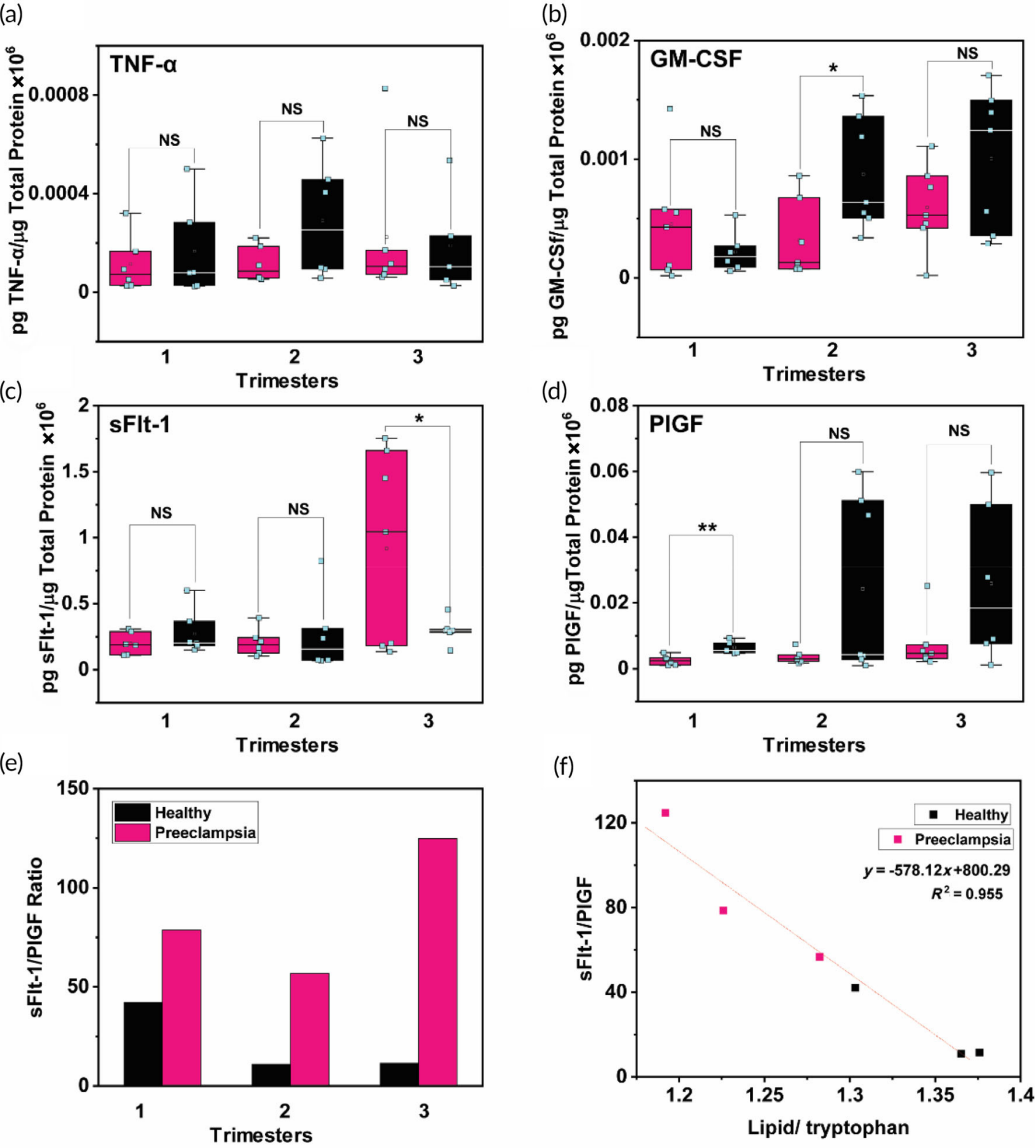
ELISA results for different proteins assessed longitudinally in all three trimesters: healthy in black and preeclampsia in pink. (a–d) Box plots showing picograms of proinflammatory cytokines TNF‐α and GMCSF, and angiogenic biomarkers sFlt‐1 and PlGF normalized to total protein concentration for *n* = 5–7 patients per cohort. (e) Average sFlt‐1/PlGF ratio in every cohort showing an increase in the anti‐angiogenic factor in preeclampsia in comparison to healthy. (f) Linear correlation of metabolites to proteins showing the ratio of lipid/tryptophan peaks to sFlt‐1/PlGF ratio in all three trimesters of healthy and preeclampsia.

We then compared metabolite levels measured with Raman to protein levels measured with enzyme linked immunosorbent assay (ELISA) of proinflammatory cytokines including tumor necrosis factor‐alpha (TNF‐α) and granulocyte‐macrophage colony‐stimulating factor (GM‐CSF), and angiogenic markers including sFlt‐1 and PlGF (Figure 6). Maternal angiogenic biomarkers including sFlt‐1 and PlGF play a crucial role in vasculogenesis and placental growth.^85^ Hypoxic environment in the placenta is associated with increase in sFlt‐1 and decrease in PlGF, and the sFlt‐1/PlGF ratio provides a measure for anti‐angiogenesis. The sFlt‐1/PlGF ratio in late 2nd trimester has shown high predictive power in predicting preeclampsia and fetal growth restriction.^10^, ^11^, ^12^ Yet in the first 20 weeks of gestation, levels of these markers in maternal blood remain inconclusive suggesting current clinical measures are inadequate in early prediction of at risk patients.^13^ Here, we performed ELISA in *n* = 5–7 healthy and preeclampsia patients; three independent experiments for each protein and duplicates for each patient was performed. Values were normalized to the total protein concentration in the samples, which we measured with bicinchoninic acid (BCA) protein assay. Our results showed no statistically significant differences in TNF‐α and GM‐CSF between healthy and preeclamptic patients in any of the trimester. These inconclusive protein levels are in part due to the poor sensitivity of ELISA that fails to measure ultra‐low levels of proteins, and also since inflammatory cytokines are known to have low positive predictive values for accurate diagnosis of preeclampsia. We also determined the sFlt‐1/PlGF ratio (Figure 6e) where for preeclamptic patients in the 1st, 2nd, and 3rd trimester the ratio was 78.6, 56.7 and 124.7 respectively, and for healthy patients the ratio was 42.1, 10.9, 11.4 in the three trimesters. Literature evidence shows a cut‐off value of ≤33 and ≥85 for early onset preeclampsia, and ≤33 and ≥110 for the late onset preeclampsia.^86^, ^87^ The dual cut‐off implies that an sFlt‐1/PlGF ratio of ≤33 had the lowest likelihood of a negative test while values ≥85 had the highest likelihood of a positive test. Our ELISA results demonstrate that in the 3rd trimester healthy patients fall below the cut‐off and preeclamptic patients above the cut‐off. However, in the healthy cohort in the 1st trimester the sFlt‐1/PlGF ratio is >33, and in the preeclamptic cohort in the 1st and 2nd trimesters the ratio is <85. These results suggest that protein analysis alone would be inadequate in ruling out the possibility of preeclampsia early in pregnancy, and other complimentary screening methodologies may be necessary.

Therefore, we also investigated if a predictive ratio could be derived from our Raman findings that could be correlated to the sFLT‐1/PlGF ratio. As lipids (956 cm^−1^) and AAs specifically tryptophan (1551 cm^−1^), were highly relevant in plasma of preeclamptic patients, we used the lipid/tryptophan ratio and found a linear correlation to sFlt‐1/PlGF ratio (Figure 6f, *R* ^2^ = 0.955). We note that since we only had three samples per patient one for each trimester, this linear curve only has 3 data points for healthy and 3 for preeclampsia. In a future clinical study, more samples per patient per trimester may improve the relationship of sFlt‐1/PlGF ratio to lipid/tryptophan ratio. Our findings imply that (i) Raman metabolic profiling can be validated with well‐established protein markers, and (ii) Raman and ELISA are not exclusive but complimentary approaches, that is protein and metabolic analysis combined together may enable a higher predictive power for diagnosing preeclampsia. We envision that a combined protein/metabolic approach could be leveraged in resource‐limited settings as Raman measurements are inexpensive with no antibody or labeling costs or costly sample preparation, and handheld Raman spectrometers that are sensitive, portable, and affordable are now commercially available. We note that our study has some limitations. Samples for this work were obtained from an academic biobank where patient biofluids are continuously banked for research and education purposes; therefore, patients were not specifically recruited for this study. Our goal was to leverage the strengths of Raman spectroscopy in metabolic analysis throughout pregnancy and enable an accurate tool to identify preeclamptic patients early in the pregnancy. We anticipate that our findings may be improved in a targeted study in future with a larger patient cohort.

## 3 CONCLUSIONS

In conclusion, this study investigates metabolic profiling with Raman spectroscopy in pregnant patient plasma throughout pregnancy, and enables rapid, low‐cost, and accurate diagnosis of patients at risk of preeclampsia. Metabolic changes within healthy and preeclamptic cohorts in all three trimesters showed distinct metabolic patterns in each cohort as patients progressed from early to late pregnancy. Raman spectral characteristics combined with tSNE analysis distinguished healthy from preeclamptic patients as early as 1st trimester. We observed multiple metabolic markers were elevated in preeclampsia including lipids, carotenoids, glucose, DNA, and citric acid while various amino acids, and protein synthesis was downregulated. KEGG analysis demonstrated that multiple pathways are altered in preeclampsia relative to healthy early and late pregnancy. These pathways are associated with various hallmarks of preeclampsia that include vascular remodeling, elevation of proinflammatory cytokines, and placental instability. A correlation analysis with the Pearson coefficient shows that metabolites have moderate to strong correlation to severity of preeclampsia throughout pregnancy. Our findings also show metabolites have correlation to other clinical and obstetric factors including BMI, maternal age, gestational age, gravida, parity, and history of hypertension. ELISA of pro‐inflammatory cytokines (TNF‐α, GM‐CSF) was not predictive for diagnosis of preeclampsia, and ratio of angiogenic markers sFlt‐1/PlGF was inadequate in the 1st and 2nd trimester in ruling out the possibility of preeclampsia but was highly accurate later in pregnancy. Our results suggest that metabolic screening throughout pregnancy will complement current prenatal care by enabling both early prediction and prognosis of the severity of preeclampsia. Further, metabolic profiling in high‐risk patients may reveal mechanisms associated with the development of preeclampsia and other prenatal diseases. On a cost perspective, in a clinical setting high risk patients undergo liver and kidney function tests, blood platelet counts, fetal ultrasound, and sFLT‐1/PlGF protein analysis among other tests. While informative, these tests are costly (depending on the healthcare provider) and not necessarily predictive of the onset of preeclampsia before the symptoms manifest. Through our approach, the only cost involved would be the capital cost of the Raman spectrometer (~$5 K for handheld Raman to ~$150 K for benchtop models) as the operation costs are relatively low. Therefore, the cost for patients would be minimal for Raman based tests. In comparison, ELISA or fluorescence based assays would require monoclonal antibodies ($500—1000/μg) and fluorophores (~$300/mg), substrates, and buffers and capital equipment ($40—70 K for a plate reader). Therefore, such tests may add to the cost for patients. Our results also show a linear correlation of sFlt‐1/PlGF ratio to lipid/tryptophan ratio suggesting metabolic and protein analysis are not exclusive but can be synergistically integrated for field analysis and in rural settings.

## 4 EXPERIMENTAL METHODS

### 4.1 Human plasma samples

All patients provided informed consent to have blood collected by the Perinatal Family Tissue Bank from the University of Iowa Hospitals & Clinics Department of Obstetrics and Gynecology (IRB#200910784). Blood was collected in ACD‐A tubes (Becton Dickinson), centrifuged, and plasma was aliquoted, snap‐frozen, and stored at −80°C. All samples and paired clinical data received from the tissue bank were coded and contained no identifying information. The number of processed samples in the healthy cohort for the 1st trimester was *n* = 23, for 2nd trimester *n* = 25 and for 3rd trimester *n* = 24. The number of processed samples in the Preeclampsia cohort for the 1st trimester was *n* = 22, for 2nd trimester *n* = 20 and for 3rd trimester *n* = 29.

### 4.2 Inclusion or exclusion criteria for collection of patient samples

Patients were not directly recruited for this study. Blood samples and clinical data were obtained from pregnant patients being recruited and enrolled by the Perinatal Family Tissue Bank (PFTB) at the University of Iowa (UI). The PFTB prospectively collects maternal biofluids (blood, urine) throughout gestation as well as at delivery (amniotic fluid, placenta). PFTB continuously recruits and enrolls participants serving as a cross sectional biobank and clinical datamart that provides research substrates for a number of studies both within UI and to other collaborating institutions. All subjects used in this study were pregnant female between the ages of 18–50 with singleton gestation. The plasma samples from the patients and associated clinical data were provided to us coded with all of the HIPAA identifiers removed. The reporting of human data aligns with the Helsinki guidelines.

### 4.3 Raman sample preparation and analysis

A Renishaw inVia Raman confocal microscope with a 785 nm laser was used to take scans of the dried plasma with WiRE 5.4 software. The spectrophotometer was calibrated daily with a standard silicon wafer at 520.5 nm. After thawing the plasma, 3 μL of sample was aliquoted on a 2 mm CaF_2_ disk and subsequently dried for 20 min in 37°C. The samples were run under the 50x objective with 100% laser power of 120 mW with 1200 lines per mm grating, 1 accumulation and 10 second exposure time. One hundred points of static scans for all the samples were measured by line maps in the WIRE 5.4 software. A custom MATLAB code (R2021b) was used to apply cosmic ray removal, baseline correction, smoothing and SNV (standard normal variate method) on the raw spectra. A Savitzky and Golay filter with a 10th order polynomial and coefficient value of 45 points was used to smooth the spectra. A 9th order polynomial with a threshold value of 0.0001 was used to remove the background fluorescence. Subsequently, all spectra were normalized to the maximum peak at 1445 ${\mathrm{cm}}^{-1}$ so that the normalized intensities ranged between 0 and 1. The peak at 1445 ${\mathrm{cm}}^{-1}$ was chosen for normalization since it showed minimal change from sample to sample. Clinical data were also normalized to values between 0 and 1. Categorical data such as severity of preeclampsia was quantified by assigning values between 0 and 3 for no preeclampsia (i.e., healthy), low, mild and severe preeclampsia (superimposed and HELLP included). These values were then also normalized between 0 and 1. Other binary features like history of preeclampsia and chronic hypertension were quantified as 0 for no and 1 for yes. Gravida, parity, maternal age and BMI were normalized to the maximum value between both cohorts. Since Raman data is high dimensional (thousands of points on the spectrum changing all at once), a robust dimensionality reduction method is needed to work with these data. T‐distributed Stochastic Neighbor Embedding (tSNE) is a non‐linear and unsupervised multivariate dimensionality reduction method. Another custom MATLAB code (R2021b) was used to show the separation between the cohorts. With tSNE of the normalized Raman spectra we can show that there is a distinction between the two groups (healthy and PE) and a clear distinction between the three trimesters within one group (see Figures 1 and 2). tSNE was used with the Exact algorithm and Cosine distance function, an exaggeration of two and varying number of PCA components and perplexity noted in the figure captions.

### 4.4 Raman spiking experiments

To confirm the Raman peak assignments, we selected a few metabolites and spiked them in one of the plasma samples from the healthy patient cohort to see if the assigned Raman peak would increase. We purchased tryptophan, citric acid, and β‐carotene powders from Sigma Aldrich. Tryptophan was dissolved in water at the starting concentration of 6 mg/mL; from this stock solution a serial dilution was made in water to achieve 3, 1.5, and 05 mg/mL solutions. These solutions were then mixed in equal volumes with plasma. From this spiked plasma, 3 μL was deposited on a CaF_2_ disk and Raman measurements were performed using the same parameters as described above. For citric acid, a starting stock of 12.8 mM (equal to 2.45 mg/mL) was made, and then a serial dilution was made in water to produce solutions with 4, 2, and 1 mM concentrations. These solutions were then mixed in equal volumes with plasma. From this spiked plasma, 3 μL was deposited on a CaF_2_ disk and Raman measurements were performed. Raman spectra of pure β‐carotene was acquired by making a 1 mg/mL solution of β‐carotene in ethanol and then drying 3 μL of the stock on a CaF_2_ disk. β‐carotene was diluted into a second stock of 40 μL/mL in ethanol, and then diluted furthermore with a 50/50 ethanol/water mixture to generate 20, 10, 5, and 2.5 μL/mL solutions. These solutions were then mixed in equal volumes with plasma. Three microliters of each spiked solution was aliquoted on a CaF_2_ disk and dried. We observed that β‐carotene did not produce a homogenous dispersion in plasma giving spot to spot variability in the plasma sample. Therefore, a standard curve could not be obtained but the spiking results in Figure S5 confirms the assigned Raman peak positions.

### 4.5 Enzyme‐linked immunosorbent assay

TNF‐α, PlGF, and sFLt‐1 ELISA kits for humans were purchased from Thermo Fisher (Waltham, MA), Invitrogen (Waltham, MA) and Bio‐Techne (Minneapolis, MN), respectively. Corning® (Corning, NY) 96 well half‐area microplates were used for TNF‐α ELISA analysis. The kits from Invitrogen and Bio‐Techne came with their own coated 96‐well microplates. Total protein was measured by BCA protein assay (Thermo Fisher 23225). The assays were performed according to the manufacturer's protocol. The ELISA concentrations were all normalized to their corresponding total protein concentrations. GraphPad Prism 8.1 software was used to read the absorbance of the well plates.

### 4.6 MetaboAnalyst

MetaboAnalyst, a web based interface for analysis of metabolic data, was used to obtain figures for AUC‐ROC analysis (Figure 2j–l), correlation analysis, enrichment analysis (Figure 4), and correlation heatmaps (Figure 5). The Biomarker Analysis module was used to produce AUC‐ROC curves in Tester mode. The peak values were uploaded for every sample in the healthy and preeclampsia cohorts. Only the strongest peak for each metabolite was used and additional peaks belonging to the same metabolite (such as phenylalanine, tryptophan, etc. has several peaks) was removed from the analysis as only a single can be used in MetaboAnalyst. We note that MetaboAnalyst offers the option to compute and add statistically significant ratios to this analysis. However, we refrained from adding this option to avoid overfitting our model. This option can be used for a much larger cohort of samples. Through this approach we achieved AUC‐ROC scores of 0.827, 0.862 and 0.926 respectively, for 1st, 2nd, and 3rd trimester. The Statistical Analysis (metadata) module was used to create correlation analysis with the Pearson r moment for RS peaks to the severity of preeclampsia. Heatmaps were generated using the Statistical Analysis (one factor) module with the default MetaboAnalyst parameters such as Pearson r distance measure. The Enrichment Analysis module with the KEGG pathway and the ID type as compound names and feature type as metabolites were used to obtain the Enrichment analysis figure. For enrichment analysis, the individual Raman peak assignments (that corresponds to the different metabolites) and the peak intensities for all of the patients in this study were uploaded into MetaboAnalyst. Here the intensity of the Raman peaks was used as a “proxy” for metabolite concentrations to obtain the corresponding metabolic pathways. This is reasonable since the Raman peak intensity is linearly correlated to the concentration of each analyte measured. Note that generic peaks such as carbohydrates (1017 cm^−1^), amino acids (1206 cm^−1^), amide III (1243 cm^−1^), amide I (1672 cm^−1^) and DNA (805, 1420 cm^−1^) were not used in the enrichment analyses as they represent a broad family instead of specific metabolites. The enrichment analyses provide *p*‐values for the most probable metabolic pathways. However, it is important to note that the enrichment *p*‐values provided by MetaboAnalyst are not the same as *p*‐values from statistical analysis. The enrichment *p*‐value is defined as the probability of obtaining 𝑛 or more pathways that give rise to a cumulative hypergeometric distribution. MetaboAnalyst selects the hypergeometric test for over‐representation analysis (ORA). ORA is a statistical method that determines whether metabolites from a pre‐defined set (such as the KEGG database) are present more than would be expected (over‐represented) in the data we upload; this represents the enrichment *p*‐values.

### 4.7 Statistical analysis

All data presented in the manuscript is shown as the standard error of the mean. For the Raman analysis we measured total 45 samples in the 1st trimester, 45 in 2nd trimester, and 53 in 3rd trimester to have biological replicates. We measured 100 data points per sample to have technical replicates. For ELISA, we measured *n* = 5–7 biological replicates and each sample was measured 3 times to have technical replicates. Unpaired, two‐sided, homoscedastic student's *t*‐tests was used to generate *p* values, where * indicates *p*‐value <0.05, **p‐value <0.01, and ***p‐value <0.001.

A power calculation on a superiority/non‐inferiority trial for this study for *n* = 45 samples/trimester gave an 80% power to detect differences between groups with a 5% type I error rate, standard deviation of 1, and minimum detectable effect of 0.5.

*Supporting information*: Clinical information tables for individual healthy and preeclampsia patients; Pearson correlation values; correlation heatmap values for each trimester; Raman metabolites associated with each KEGG metabolic pathway; Raman spectra of individual healthy and preeclampsia patients in each trimester; box plots of additional Raman metabolites; and metabolite spiking experiments to confirm Raman peaks. This material is available free of charge via the Internet https://www.aiche.org/publications/journals/bioengineering-translational-medicine

## AUTHOR CONTRIBUTIONS

**Saman Ghazvini:** Investigation (lead); methodology (lead); writing – original draft (lead). **Saji Uthaman:** Formal analysis (supporting); investigation (supporting); writing – review and editing (supporting). **Lilly Synan:** Methodology (supporting). **Eugene C. Lin:** Software (equal). **Soumik Sarkar:** Software (equal); writing – review and editing (supporting). **Mark K. Santillan:** Data curation (supporting); writing – review and editing (supporting). **Donna A. Santillan:** Data curation (supporting); writing – review and editing (supporting). **Rizia Bardhan:** Conceptualization (lead); supervision (lead).

## CONFLICT OF INTEREST STATEMENT

The authors declare no conflicts of interest.

## Supporting information


**Data S1.** Supplementary Information.

## Data Availability

The data supporting this study's findings are available from the corresponding author upon reasonable request.
