# Supplementary material for: Predicting the onset of preeclampsia by longitudinal monitoring of metabolic changes throughout pregnancy with Raman spectroscopy
Source: Bioeng Transl Med. 2023 Aug 31;9(1):e10595. doi: 10.1002/btm2.10595 (PMC10771567; doi:10.1002/btm2.10595)
Supplement: Supplementary file 1 — Data S1. Supplementary Information. [file BTM2-9-e10595-s001.docx]

**Supporting Information**

**Predicting the onset of preeclampsia by longitudinal monitoring of metabolic changes throughout pregnancy with Raman spectroscopy**

Saman Ghazvini^a,b^, Saji Uthaman^a,b^, Lilly Synan^a,b^, Eugene C. Lin^c^, Soumik Sarkar^d^, Mark K. Santillan^e^, Donna A. Santillan^e^, Rizia Bardhan^a,b,^*

^a^Department of Chemical and Biological Engineering, Iowa State University, Ames, IA 50012, USA

^b^Nanovaccine Institute, Iowa State University, Ames, IA 50012, USA

^c^Department of Chemistry and Biochemistry, National Chung Cheng University, Chiayi 62106, Taiwan

^d^Department of Mechanical Engineering, Iowa state University, Ames, IA 50012, USA

^e^Department of Obstetrics and Gynecology, Carver College of Medicine, University of Iowa, Hospitals & Clinics, Iowa City, IA 52242, USA

*Corresponding author: [rbardhan@iastate.edu](mailto:rbardhan@iastate.edu)

**Table S1.** Healthy patients’ clinical information including maternal age, gestational age at delivery, body mass index (BMI), number of pregnancies (gravida), number of births (parity), number of pregnancy losses, chronic hypertension, current hypertension, history of preeclampsia (PE), and severity of PE.

| **Patient #** | **Maternal Age (years)** | **Gestational age (weeks)** | **BMI (kg/m²)** | **Gravida** | **Parity** | **Pregnancy loss** | **Chronic hypertension** | **Current hypertension** | **History of PE** | **Severity of PE** |
| --- | --- | --- | --- | --- | --- | --- | --- | --- | --- | --- |
| 1 | 35 | 40.57 | 21.5 | 3 | 2 | 0 | 0 | 0 | 0 | None |
| 2 | 32 | 41.29 | 23.84 | 2 | 1 | 0 | 0 | 0 | 0 | None |
| 3 | 33 | 39.29 | 18 | 8 | 4 | 3 | 0 | 0 | 0 | None |
| 4 | 41 | 39.00 | 53.0 | 3 | 2 | 0 | 1 | 0 | 0 | None |
| 5 | 30 | 39.57 | 20.72 | 2 | 1 | 0 | 0 | 0 | 0 | None |
| 6 | 34 | 38.14 | 27.43 | 2 | 1 | 0 | 0 | 1 | 0 | None |
| 7 | 35 | 39.00 | 26.98 | 5 | 3 | 1 | 0 | 0 | 0 | None |
| 8 | 31 | 39.57 | 25.72 | 2 | 1 | 0 | 0 | 0 | 0 | None |
| 9 | 30 | 35.71 | 30.01 | 2 | 1 | 0 | 0 | 0 | 0 | None |
| 10 | 25 | 38.43 | 35.4 | 2 | 1 | 0 | 0 | 0 | 0 | None |
| 11 | 33 | 40.86 | 18.9 | 1 | 0 | 0 | 0 | 0 | 0 | None |
| 12 | 36 | 39.43 | 20.21 | 1 | 1 | 0 | 0 | 0 | 0 | None |
| 13 | 31 | 37.14 | 52.07 | 7 | 2 | 4 | 1 | 0 | 1 | None |
| 14 | 31 | 39.00 | 18.85 | 2 | 1 | 0 | 0 | 0 | 0 | None |
| 15 | 36 | 37.00 | 34.4 | 2 | 1 | 0 | 1 | 0 | 0 | None |
| 16 | 23 | 38.14 | 36.36 | 3 | 2 | 0 | 0 | 0 | 0 | None |
| 17 | 19 | 39.57 | 21.6 | 1 | 0 | 0 | 0 | 0 | 0 | None |
| 18 | 29 | 39.00 | 24.09 | 4 | 2 | 1 | 0 | 0 | 0 | None |
| 19 | 28 | 34.43 | 26.5 | 3 | 1 | 1 | 0 | 1 | 0 | None |
| 20 | 25 | 37.29 | 22.2 | 6 | 2 | 2 | 0 | 0 | 0 | None |
| 21 | 33 | 37.00 | 22.2 | 2 | 1 | 0 | 0 | 0 | 0 | None |
| 22 | 38 | 40.71 | 21.89 | 3 | 1 | 1 | 0 | 0 | 0 | None |
| 23 | 30 | 32.00 | 51.3 | 5 | 2 | 2 | 0 | 0 | 0 | None |
| 24 | 31 | 39.86 | 33.64 | 3 | 2 | 1 | 0 | 0 | 0 | None |
| 25 | 30 | 39.00 | 48.95 | 2 | 1 | 0 | 0 | 0 | 0 | None |
| 26 | 37 | 39.14 | 27.12 | 2 | 1 | 0 | 0 | 0 | 0 | None |
| 27 | 25 | 38.57 | 45.15 | 1 | 0 | 0 | 0 | 1 | 0 | None |
| 28 | 31 | 39.14 | 59.56 | 1 | 0 | 0 | 0 | 1 | 0 | None |
| 29 | 33 | 34.71 | 23.24 | 1 | 0 | 0 | 0 | 0 | 0 | None |
| 30 | 31 | 39.14 | 31.24 | 2 | 1 | 0 | 0 | 0 | 0 | None |
| 31 | 29 | 38.43 | 34.88 | 1 | 0 | 0 | 1 | 0 | 0 | None |
| 32 | 29 | 39.14 | 34.88 | 1 | 0 | 0 | 1 | 0 | 0 | No |
| 33 | 21 | 40.57 | 24.19 | 2 | 1 | 0 | 0 | 0 | 0 | No |
| 34 | 33 | 38.14 | 32.91 | 2 | 0 | 1 | 0 | 0 | 0 | No |

**Table S2.** Preeclamptic patients’ clinical information including maternal age, gestational age at delivery, body mass index (BMI), number of pregnancies (gravida), number of births (parity), number of pregnancy losses, chronic hypertension, current hypertension, history of preeclampsia (PE), and severity of PE.

| **Patient #** | **Maternal Age (years)** | **Gestational age (weeks)** | **BMI (kg/m²)** | **Gravida** | **Parity** | **Pregnancy loss** | **Chronic hypertension** | **Current hypertension** | **History of PE** | **Severity of PE** |
| --- | --- | --- | --- | --- | --- | --- | --- | --- | --- | --- |
| 1 | 26 | 34.29 | 27.71 | 2 | 0 | 0 | 0 | 0 | 0 | HELLP |
| 2 | 35 | 34.29 | 20.59 | 5 | 3 | 1 | 0 | 0 | 1 | Severe |
| 3 | 29 | 39.86 | 29.44 | 2 | 1 | 0 | 0 | 0 | 0 | Severe |
| 4 | 27 | 37.29 | 24.76 | 1 | 0 | 0 | 0 | 0 | 0 | HELLP |
| 5 | 32 | 38.57 | 24.78 | 2 | 1 | 0 | 0 | 0 | 1 | Mild |
| 6 | 29 | 38.43 | 29.7 | 1 | 0 | 0 | 1 | 0 | 0 | Superimposed - severe |
| 7 | 31 | 39.29 | 33.99 | 2 | 1 | 0 | 1 | 0 | 0 | Superimposed - severe |
| 8 | 20 | 36.00 | 30.16 | 2 | 1 | 0 | 0 | 0 | 1 | Mild |
| 9 | 30 | 37.86 | 36.05 | 5 | 2 | 2 | 1 | 1 | 0 | Superimposed - severe |
| 10 | 37 | 37.43 | 31.1 | 3 | 1 | 0 | 0 | 0 | 0 | Severe |
| 11 | 34 | 31.29 | 21.95 | 7 | 3 | 1 | 1 | 0 | 1 | Superimposed - severe |
| 12 | 28 | 34.43 | 26.45 | 3 | 1 | 0 | 0 | 1 | 0 | Severe |
| 13 | 39 | 36.57 | 37.73 | 7 | 1 | 3 | 1 | 0 | 0 | Superimposed - severe |
| 14 | 42 | 36.29 | 35.12 | 2 | 1 | 0 | 0 | 0 | 1 | Mild |
| 15 | 50 | 36.43 | 21.37 | 7 | 1 | 4 | 0 | 1 | 0 | Severe |
| 16 | 26 | 38.57 | 25.06 | 2 | 1 | 0 | 0 | 0 | 0 | Mild |
| 17 | 35 | 35.00 | 29.18 | 4 | 2 | 1 | 1 | 0 | 0 | Superimposed - severe |
| 18 | 35 | 37.29 | 26.87 | 1 | 0 | 0 | 1 | 0 | 0 | Mild |
| 19 | 36 | 35.29 | 52.97 | 1 | 0 | 0 | 0 | 0 | 0 | Severe |
| 20 | 23 | 37.29 | 25.29 | 2 | 1 | 0 | 0 | 0 | 0 | Mild |
| 21 | 33 | 34.86 | 25.51 | 1 | 0 | 0 | 0 | 0 | 0 | Mild |
| 22 | 30 | 37.14 | 29.59 | 1 | 0 | 0 | 0 | 0 | 0 | Mild |
| 23 | 29 | 41.14 | 33.8 | 1 | 0 | 0 | 0 | 0 | 0 | Postpartum Severe |
| 24 | 26 | 37.29 | 20.93 | 1 | 0 | 0 | 0 | 0 | 0 | Mild |
| 25 | 30 | 34.71 | 21.93 | 1 | 0 | 0 | 0 | 0 | 0 | Severe |
| 26 | 29 | 37.14 | 45.97 | 1 | 0 | 0 | 0 | 1 | 0 | Severe |
| 27 | 39 | 35.14 | 33.34 | 3 | 1 | 0 | 0 | 0 | 0 | Severe |
| 28 | 45 | 34.29 | 23.7 | 2 | 0 | 1 | 0 | 0 | 0 | HELLP |
| 29 | 24 | 38.00 | 31.45 | 5 | 1 | 3 | 0 | 0 | 0 | Mild |
| 30 | 30 | 30.57 | 26.01 | 2 | 1 | 0 | 0 | 0 | 0 | Severe |
| 31 | 26 | 39.86 | 25.46 | 1 | 0 | 0 | 0 | 0 | 0 | Mild |
| 32 | 35 | 31.14 | 21.61 | 2 | 1 | 0 | 0 | 0 | 1 | Mild |
| 33 | 29 | 35.00 | 21.93 | 1 | 0 | 0 | 0 | 0 | 0 | Mild |
| 34 | 31 | 31.57 | 29.3 | 1 | 0 | 0 | 0 | 0 | 0 | HELLP |
| 35 | 31 | 30.86 | 23.11 | 1 | 0 | 0 | 0 | 0 | 0 | Severe |
| 36 | 32 | 40.43 | 22.86 | 1 | 0 | 0 | 0 | 0 | 0 | Mild |
| 37 | 39 | 36.00 | 31.51 | 5 | 4 | 0 | 0 | 0 | 1 | Mild |
| 38 | 32 | 37.14 | 21.43 | 1 | 0 | 0 | 0 | 0 | 0 | Mild |
| 39 | 34 | 37.29 | 34.34 | 4 | 2 | 1 | 1 | 0 | 1 | Superimposed - severe |
| 40 | 35 | 37.86 | 20.39 | 5 | 4 | 0 | 0 | 0 | 1 | Mild |

**Table S3.** Pearson correlation values of Raman peaks to the severity of preeclampsia in the 1^st^ trimester associated with Figure 5a. PC/PE: phosphatidylcholine / phosphatidylethanolamine.

| **Wavenumber (cm^-1^)** | **Metabolite** | **Correlation ratio** |
| --- | --- | --- |
| 940 | Citric/Succinic acid | 0.51614 |
| 898 | Glycine | 0.51225 |
| 1517 | Carotenoids | 0.51081 |
| 1154 | Carotenoids | 0.50061 |
| 1243 | Amide III | -0.37833 |
| 701 | Cholesterol | 0.37749 |
| 1551 | Tryptophan | -0.35051 |
| 1017 | Carbohydrates | -0.33882 |
| 1657 | Unsaturated lipids, PC/PE | 0.32267 |
| 956 | Lipids | 0.27921 |
| 848 | Sugars | 0.26096 |
| 1340 | Threonine | 0.25443 |
| 987 | Arginine | -0.19636 |

**Table S4.** Pearson correlation values of Raman peaks to the severity of preeclampsia in the 2^nd^ trimester associated with Figure 5b. PC/PE: phosphatidylcholine / phosphatidylethanolamine.

| **Wavenumber (cm^-1^)** | **Metabolite** | **Correlation ratio** |
| --- | --- | --- |
| 1206 | Amino acids | -0.63194 |
| 1243 | Amide III | -0.60527 |
| 1672 | Amide I | -0.5625 |
| 1265 | Unsaturated lipids, fatty acids | -0.49046 |
| 1551 | Tryptophan | -0.43228 |
| 1420 | DNA | 0.38694 |
| 956 | Lipids | 0.38191 |
| 1154 | Carotenoids | 0.37569 |
| 1017 | Carbohydrates | -0.37537 |
| 1317 | Histidine | -0.3748 |
| 1657 | Unsaturated lipids, PC/PE | -0.3702 |
| 987 | Arginine | -0.35756 |
| 1031 | Phenylalanine | -0.34739 |
| 835 | Tyrosine | 0.34195 |
| 1517 | Carotenoids | 0.32829 |
| 1340 | Threonine | -0.28515 |
| 848 | Sugars | 0.28039 |
| 940 | Citric/Succinic acid | 0.24798 |

**Table S5.** Pearson correlation values of Raman peaks to the severity of preeclampsia in the 3^rd^ trimester associated with Figure 5c.

| **Wavenumber (cm^-1^)** | **Metabolite** | **Correlation ratio** |
| --- | --- | --- |
| 1243 | Amide III | -0.58089 |
| 1154 | Carotenoids | 0.57551 |
| 956 | Lipids | 0.56053 |
| 1517 | Carotenoids | 0.54239 |
| 1551 | Tryptophan | -0.5001 |
| 848 | Sugars | 0.46076 |
| 1584 | Phenylalanine | 0.45683 |
| 1206 | Amino acids | -0.44618 |
| 1017 | Carbohydrates | -0.43235 |
| 1031 | Phenylalanine | -0.38299 |
| 1317 | Histidine | -0.36165 |
| 940 | Citric/Succinic acid | 0.35228 |
| 1420 | DNA | 0.34755 |
| 1340 | Threonine | -0.33235 |
| 1672 | Amide I | -0.30078 |
| 1303 | Triglycerides | -0.30059 |
| 898 | Glycine | 0.29481 |
| 835 | Tyrosine | 0.24902 |
| 757 | Tryptophan | -0.23907 |

**Table S6.** Correlation coefficient values for Raman data and clinical information heat map in 1^st^ trimester associated with Figure 5d. Red, yellow, and green cells stand for weak, moderate, and strong correlation levels respectively. Gray cells are direct comparisons of a feature to itself. Values between ± (0 - 0.29) suggest a weak correlation, ± (0.30 - 0.50) moderate correlation, and ± (0.51 - 1) suggest strong correlation of variates to each other. Negative values indicate inverse correlation, and positive values indicate joint correlation. Abbreviations used: BMI: body mass index, Ch Hyp: Chronic hypertension, Cu Hyp: Current hypertension, GA: gestational age, MA: maternal age, Gr: gravida, Pa: parity, HoP: history of preeclampsia, SoP: severity of preeclampsia, PL: pregnancy loss.

|  | GA | BMI | Cu. Hyp. | 987 | 1551 | 1017 | 1243 | 1254 | 701 | 681 | 898 | 940 | 848 | 956 | 1420 | 1657 | 1154 | 1517 | 1340 | MA | Ch. Hyp. | Gr | Pa | HoP | PL |
| --- | --- | --- | --- | --- | --- | --- | --- | --- | --- | --- | --- | --- | --- | --- | --- | --- | --- | --- | --- | --- | --- | --- | --- | --- | --- |
| GA | 1.00 | 0.15 | 0.00 | -0.03 | 0.27 | 0.29 | 0.25 | 0.16 | -0.09 | 0.02 | -0.04 | -0.13 | 0.04 | -0.16 | 0.04 | -0.37 | -0.37 | -0.32 | -0.10 | -0.07 | -0.08 | -0.43 | -0.24 | -0.29 | -0.22 |
| BMI | 0.15 | 1.00 | 0.35 | 0.27 | 0.14 | 0.26 | 0.33 | 0.38 | -0.07 | -0.21 | -0.23 | -0.15 | -0.08 | -0.06 | -0.01 | -0.21 | -0.35 | -0.38 | -0.12 | -0.03 | 0.22 | 0.03 | -0.07 | -0.02 | 0.21 |
| Cu. Hyp. | 0.00 | 0.35 | 1.00 | 0.19 | -0.01 | 0.21 | 0.12 | 0.14 | 0.04 | 0.08 | 0.09 | 0.11 | -0.11 | 0.02 | -0.02 | -0.14 | -0.08 | -0.10 | -0.09 | -0.23 | -0.17 | -0.16 | -0.21 | -0.16 | -0.03 |
| 987 | -0.03 | 0.27 | 0.19 | 1.00 | 0.09 | 0.19 | 0.28 | 0.42 | -0.22 | -0.47 | -0.53 | -0.48 | -0.30 | -0.49 | -0.32 | 0.07 | -0.15 | -0.15 | -0.14 | -0.14 | -0.06 | -0.01 | -0.01 | -0.08 | -0.19 |
| 1551 | 0.27 | 0.14 | -0.01 | 0.09 | 1.00 | 0.49 | 0.56 | 0.43 | -0.27 | -0.13 | -0.31 | -0.39 | -0.09 | -0.36 | -0.14 | -0.27 | -0.52 | -0.50 | 0.05 | 0.17 | 0.13 | -0.12 | -0.06 | -0.19 | -0.26 |
| 1017 | 0.29 | 0.26 | 0.21 | 0.19 | 0.49 | 1.00 | 0.66 | 0.64 | 0.00 | -0.10 | -0.10 | -0.10 | 0.09 | -0.07 | 0.01 | -0.28 | -0.36 | -0.43 | -0.15 | 0.02 | 0.06 | -0.28 | -0.17 | -0.29 | -0.31 |
| 1243 | 0.25 | 0.33 | 0.12 | 0.28 | 0.56 | 0.66 | 1.00 | 0.92 | -0.29 | -0.34 | -0.35 | -0.36 | -0.29 | -0.35 | -0.20 | -0.17 | -0.44 | -0.45 | 0.14 | -0.09 | 0.20 | -0.02 | 0.03 | -0.06 | -0.06 |
| 1254 | 0.16 | 0.38 | 0.14 | 0.42 | 0.43 | 0.64 | 0.92 | 1.00 | -0.19 | -0.33 | -0.38 | -0.35 | -0.27 | -0.36 | -0.26 | -0.09 | -0.42 | -0.42 | 0.04 | -0.11 | 0.22 | -0.06 | -0.02 | -0.15 | -0.11 |
| 701 | -0.09 | -0.07 | 0.04 | -0.22 | -0.27 | 0.00 | -0.29 | -0.19 | 1.00 | 0.74 | 0.72 | 0.68 | 0.34 | 0.55 | 0.18 | -0.10 | 0.30 | 0.26 | 0.00 | -0.28 | -0.08 | -0.06 | -0.09 | 0.10 | -0.03 |
| 681 | 0.02 | -0.21 | 0.08 | -0.47 | -0.13 | -0.10 | -0.34 | -0.33 | 0.74 | 1.00 | 0.86 | 0.79 | 0.30 | 0.68 | 0.44 | -0.17 | 0.12 | 0.13 | 0.06 | -0.12 | -0.03 | -0.18 | -0.25 | -0.02 | 0.00 |
| 898 | -0.04 | -0.23 | 0.09 | -0.53 | -0.31 | -0.10 | -0.35 | -0.38 | 0.72 | 0.86 | 1.00 | 0.96 | 0.32 | 0.77 | 0.46 | -0.23 | 0.29 | 0.24 | 0.18 | -0.15 | -0.05 | -0.16 | -0.17 | 0.11 | 0.08 |
| 940 | -0.13 | -0.15 | 0.11 | -0.48 | -0.39 | -0.10 | -0.36 | -0.35 | 0.68 | 0.79 | 0.96 | 1.00 | 0.32 | 0.80 | 0.49 | -0.21 | 0.29 | 0.20 | 0.22 | -0.18 | -0.02 | -0.16 | -0.19 | 0.09 | 0.08 |
| 848 | 0.04 | -0.08 | -0.11 | -0.30 | -0.09 | 0.09 | -0.29 | -0.27 | 0.34 | 0.30 | 0.32 | 0.32 | 1.00 | 0.30 | 0.20 | -0.18 | 0.17 | 0.11 | -0.01 | 0.04 | 0.09 | -0.20 | -0.26 | -0.14 | 0.01 |
| 956 | -0.16 | -0.06 | 0.02 | -0.49 | -0.36 | -0.07 | -0.35 | -0.36 | 0.55 | 0.68 | 0.77 | 0.80 | 0.30 | 1.00 | 0.80 | -0.39 | 0.21 | 0.06 | -0.14 | -0.14 | -0.13 | -0.09 | -0.11 | 0.04 | 0.03 |
| 1420 | 0.04 | -0.01 | -0.02 | -0.32 | -0.14 | 0.01 | -0.20 | -0.26 | 0.18 | 0.44 | 0.46 | 0.49 | 0.20 | 0.80 | 1.00 | -0.52 | -0.16 | -0.29 | -0.32 | -0.02 | -0.15 | -0.20 | -0.17 | -0.08 | -0.05 |
| 1657 | -0.37 | -0.21 | -0.14 | 0.07 | -0.27 | -0.28 | -0.17 | -0.09 | -0.10 | -0.17 | -0.23 | -0.21 | -0.18 | -0.39 | -0.52 | 1.00 | 0.49 | 0.60 | 0.17 | 0.06 | 0.08 | 0.15 | 0.14 | 0.08 | -0.03 |
| 1154 | -0.37 | -0.35 | -0.08 | -0.15 | -0.52 | -0.36 | -0.44 | -0.42 | 0.30 | 0.12 | 0.29 | 0.29 | 0.17 | 0.21 | -0.16 | 0.49 | 1.00 | 0.96 | 0.19 | -0.10 | -0.08 | 0.15 | 0.25 | 0.32 | 0.04 |
| 1517 | -0.32 | -0.38 | -0.10 | -0.15 | -0.50 | -0.43 | -0.45 | -0.42 | 0.26 | 0.13 | 0.24 | 0.20 | 0.11 | 0.06 | -0.29 | 0.60 | 0.96 | 1.00 | 0.20 | -0.02 | -0.04 | 0.16 | 0.27 | 0.33 | 0.07 |
| 1340 | -0.10 | -0.12 | -0.09 | -0.14 | 0.05 | -0.15 | 0.14 | 0.04 | 0.00 | 0.06 | 0.18 | 0.22 | -0.01 | -0.14 | -0.32 | 0.17 | 0.19 | 0.20 | 1.00 | -0.05 | 0.30 | 0.23 | 0.21 | 0.33 | 0.24 |
| MA | -0.07 | -0.03 | -0.23 | -0.14 | 0.17 | 0.02 | -0.09 | -0.11 | -0.28 | -0.12 | -0.15 | -0.18 | 0.04 | -0.14 | -0.02 | 0.06 | -0.10 | -0.02 | -0.05 | 1.00 | 0.27 | 0.17 | 0.22 | 0.12 | 0.13 |
| Ch. Hyp. | -0.08 | 0.22 | -0.17 | -0.06 | 0.13 | 0.06 | 0.20 | 0.22 | -0.08 | -0.03 | -0.05 | -0.02 | 0.09 | -0.13 | -0.15 | 0.08 | -0.08 | -0.04 | 0.30 | 0.27 | 1.00 | 0.37 | 0.32 | 0.28 | 0.43 |
| Gr | -0.43 | 0.03 | -0.16 | -0.01 | -0.12 | -0.28 | -0.02 | -0.06 | -0.06 | -0.18 | -0.16 | -0.16 | -0.20 | -0.09 | -0.20 | 0.15 | 0.15 | 0.16 | 0.23 | 0.17 | 0.37 | 1.00 | 0.85 | 0.57 | 0.59 |
| Pa | -0.24 | -0.07 | -0.21 | -0.01 | -0.06 | -0.17 | 0.03 | -0.02 | -0.09 | -0.25 | -0.17 | -0.19 | -0.26 | -0.11 | -0.17 | 0.14 | 0.25 | 0.27 | 0.21 | 0.22 | 0.32 | 0.85 | 1.00 | 0.60 | 0.31 |
| HoP | -0.29 | -0.02 | -0.16 | -0.08 | -0.19 | -0.29 | -0.06 | -0.15 | 0.10 | -0.02 | 0.11 | 0.09 | -0.14 | 0.04 | -0.08 | 0.08 | 0.32 | 0.33 | 0.33 | 0.12 | 0.28 | 0.57 | 0.60 | 1.00 | 0.47 |
| PL | -0.22 | 0.21 | -0.03 | -0.19 | -0.26 | -0.31 | -0.06 | -0.11 | -0.03 | 0.00 | 0.08 | 0.08 | 0.01 | 0.03 | -0.05 | -0.03 | 0.04 | 0.07 | 0.24 | 0.13 | 0.43 | 0.59 | 0.31 | 0.47 | 1.00 |

**Table S7.** Correlation coefficient values for Raman data and clinical information heat map in 2^nd^ trimester associated with Figure 5e.

|  | 1206 | 1551 | 1017 | 1031 | 1317 | 1340 | 876 | 987 | 1265 | 1243 | 1672 | BMI | Cu. Hyp | 1657 | GA | 1420 | 940 | 956 | 835 | 848 | Ch. Hyp. | Pa | HoP | 1154 | 1517 | MA | Gr | PL |
| --- | --- | --- | --- | --- | --- | --- | --- | --- | --- | --- | --- | --- | --- | --- | --- | --- | --- | --- | --- | --- | --- | --- | --- | --- | --- | --- | --- | --- |
| 1206 | 1.00 | 0.74 | 0.51 | 0.63 | 0.61 | 0.70 | 0.23 | 0.57 | 0.32 | 0.75 | 0.53 | 0.07 | -0.04 | 0.13 | 0.31 | -0.21 | 0.01 | -0.20 | -0.22 | -0.16 | -0.17 | -0.18 | -0.31 | -0.46 | -0.45 | -0.49 | -0.49 | -0.51 |
| 1551 | 0.74 | 1.00 | 0.62 | 0.56 | 0.28 | 0.34 | 0.26 | 0.29 | 0.37 | 0.75 | 0.50 | 0.26 | 0.09 | 0.03 | 0.25 | -0.16 | -0.13 | -0.26 | -0.13 | -0.13 | -0.20 | -0.32 | -0.43 | -0.45 | -0.42 | -0.41 | -0.50 | -0.44 |
| 1017 | 0.51 | 0.62 | 1.00 | 0.86 | -0.07 | 0.02 | 0.18 | 0.19 | -0.07 | 0.33 | 0.24 | 0.19 | 0.13 | -0.01 | 0.26 | 0.24 | 0.18 | 0.13 | 0.20 | 0.19 | -0.18 | -0.25 | -0.41 | -0.50 | -0.52 | -0.17 | -0.23 | -0.10 |
| 1031 | 0.63 | 0.56 | 0.86 | 1.00 | 0.07 | 0.23 | 0.05 | 0.43 | -0.18 | 0.22 | 0.02 | 0.15 | 0.05 | -0.09 | 0.21 | 0.41 | 0.47 | 0.37 | 0.33 | 0.36 | -0.21 | -0.24 | -0.29 | -0.58 | -0.61 | -0.41 | -0.30 | -0.22 |
| 1317 | 0.61 | 0.28 | -0.07 | 0.07 | 1.00 | 0.95 | 0.51 | 0.68 | 0.38 | 0.43 | 0.41 | -0.14 | -0.22 | 0.27 | 0.21 | -0.62 | -0.04 | -0.34 | -0.56 | -0.41 | 0.15 | 0.04 | 0.10 | 0.07 | 0.15 | -0.21 | -0.26 | -0.33 |
| 1340 | 0.70 | 0.34 | 0.02 | 0.23 | 0.95 | 1.00 | 0.40 | 0.74 | 0.20 | 0.39 | 0.33 | -0.19 | -0.21 | 0.12 | 0.16 | -0.41 | 0.15 | -0.14 | -0.38 | -0.24 | 0.07 | 0.00 | 0.12 | 0.03 | 0.09 | -0.31 | -0.35 | -0.46 |
| 876 | 0.23 | 0.26 | 0.18 | 0.05 | 0.51 | 0.40 | 1.00 | 0.54 | 0.50 | 0.17 | 0.19 | -0.21 | 0.04 | 0.15 | 0.22 | -0.55 | 0.15 | -0.03 | -0.08 | 0.06 | -0.10 | -0.02 | -0.12 | 0.42 | 0.47 | -0.07 | -0.03 | 0.08 |
| 987 | 0.57 | 0.29 | 0.19 | 0.43 | 0.68 | 0.74 | 0.54 | 1.00 | 0.16 | 0.18 | -0.02 | -0.24 | -0.20 | -0.12 | 0.20 | -0.22 | 0.47 | 0.21 | 0.04 | 0.19 | -0.08 | 0.02 | 0.10 | 0.01 | 0.03 | -0.41 | -0.24 | -0.33 |
| 1265 | 0.32 | 0.37 | -0.07 | -0.18 | 0.38 | 0.20 | 0.50 | 0.16 | 1.00 | 0.68 | 0.43 | 0.21 | 0.10 | 0.21 | 0.19 | -0.71 | -0.42 | -0.51 | -0.35 | -0.30 | -0.10 | -0.05 | -0.26 | 0.11 | 0.12 | -0.20 | -0.16 | -0.12 |
| 1243 | 0.75 | 0.75 | 0.33 | 0.22 | 0.43 | 0.39 | 0.17 | 0.18 | 0.68 | 1.00 | 0.71 | 0.28 | 0.00 | 0.12 | 0.25 | -0.52 | -0.53 | -0.64 | -0.46 | -0.47 | -0.11 | -0.12 | -0.36 | -0.41 | -0.39 | -0.29 | -0.38 | -0.42 |
| 1672 | 0.53 | 0.50 | 0.24 | 0.02 | 0.41 | 0.33 | 0.19 | -0.02 | 0.43 | 0.71 | 1.00 | 0.16 | 0.11 | 0.65 | 0.41 | -0.54 | -0.65 | -0.76 | -0.74 | -0.74 | 0.05 | 0.00 | -0.33 | -0.20 | -0.13 | 0.03 | -0.20 | -0.24 |
| BMI | 0.07 | 0.26 | 0.19 | 0.15 | -0.14 | -0.19 | -0.21 | -0.24 | 0.21 | 0.28 | 0.16 | 1.00 | 0.34 | 0.15 | -0.01 | 0.00 | -0.27 | -0.24 | -0.15 | -0.20 | 0.23 | -0.27 | -0.18 | -0.31 | -0.32 | 0.03 | -0.26 | -0.18 |
| Cu. Hyp. | -0.04 | 0.09 | 0.13 | 0.05 | -0.22 | -0.21 | 0.04 | -0.20 | 0.10 | 0.00 | 0.11 | 0.34 | 1.00 | 0.27 | -0.09 | 0.10 | 0.02 | 0.06 | 0.04 | 0.04 | -0.19 | -0.18 | -0.15 | 0.15 | 0.13 | 0.08 | -0.02 | 0.12 |
| 1657 | 0.13 | 0.03 | -0.01 | -0.09 | 0.27 | 0.12 | 0.15 | -0.12 | 0.21 | 0.12 | 0.65 | 0.15 | 0.27 | 1.00 | 0.36 | -0.29 | -0.34 | -0.45 | -0.65 | -0.60 | 0.12 | -0.02 | -0.21 | 0.00 | 0.09 | 0.20 | -0.01 | 0.07 |
| GA | 0.31 | 0.25 | 0.26 | 0.21 | 0.21 | 0.16 | 0.22 | 0.20 | 0.19 | 0.25 | 0.41 | -0.01 | -0.09 | 0.36 | 1.00 | -0.21 | -0.14 | -0.23 | -0.23 | -0.17 | -0.18 | -0.14 | -0.34 | -0.05 | -0.02 | -0.11 | -0.30 | -0.13 |
| 1420 | -0.21 | -0.16 | 0.24 | 0.41 | -0.62 | -0.41 | -0.55 | -0.22 | -0.71 | -0.52 | -0.54 | 0.00 | 0.10 | -0.29 | -0.21 | 1.00 | 0.61 | 0.76 | 0.72 | 0.65 | -0.18 | -0.14 | 0.03 | -0.24 | -0.31 | -0.12 | 0.03 | 0.08 |
| 940 | 0.01 | -0.13 | 0.18 | 0.47 | -0.04 | 0.15 | 0.15 | 0.47 | -0.42 | -0.53 | -0.65 | -0.27 | 0.02 | -0.34 | -0.14 | 0.61 | 1.00 | 0.94 | 0.73 | 0.82 | -0.23 | -0.12 | 0.16 | 0.18 | 0.13 | -0.31 | -0.06 | -0.02 |
| 956 | -0.20 | -0.26 | 0.13 | 0.37 | -0.34 | -0.14 | -0.03 | 0.21 | -0.51 | -0.64 | -0.76 | -0.24 | 0.06 | -0.45 | -0.23 | 0.76 | 0.94 | 1.00 | 0.88 | 0.92 | -0.24 | -0.07 | 0.17 | 0.19 | 0.11 | -0.21 | 0.06 | 0.09 |
| 835 | -0.22 | -0.13 | 0.20 | 0.33 | -0.56 | -0.38 | -0.08 | 0.04 | -0.35 | -0.46 | -0.74 | -0.15 | 0.04 | -0.65 | -0.23 | 0.72 | 0.73 | 0.88 | 1.00 | 0.98 | -0.32 | -0.11 | 0.04 | 0.09 | -0.02 | -0.27 | 0.05 | 0.11 |
| 848 | -0.16 | -0.13 | 0.19 | 0.36 | -0.41 | -0.24 | 0.06 | 0.19 | -0.30 | -0.47 | -0.74 | -0.20 | 0.04 | -0.60 | -0.17 | 0.65 | 0.82 | 0.92 | 0.98 | 1.00 | -0.33 | -0.10 | 0.04 | 0.17 | 0.06 | -0.31 | 0.04 | 0.12 |
| Ch. Hyp | -0.17 | -0.20 | -0.18 | -0.21 | 0.15 | 0.07 | -0.10 | -0.08 | -0.10 | -0.11 | 0.05 | 0.23 | -0.19 | 0.12 | -0.18 | -0.18 | -0.23 | -0.24 | -0.32 | -0.33 | 1.00 | 0.07 | 0.20 | -0.01 | 0.03 | 0.26 | 0.16 | 0.10 |
| Pa | -0.18 | -0.32 | -0.25 | -0.24 | 0.04 | 0.00 | -0.02 | 0.02 | -0.05 | -0.12 | 0.00 | -0.27 | -0.18 | -0.02 | -0.14 | -0.14 | -0.12 | -0.07 | -0.11 | -0.10 | 0.07 | 1.00 | 0.56 | 0.16 | 0.17 | 0.23 | 0.78 | 0.39 |
| HoP | -0.31 | -0.43 | -0.41 | -0.29 | 0.10 | 0.12 | -0.12 | 0.10 | -0.26 | -0.36 | -0.33 | -0.18 | -0.15 | -0.21 | -0.34 | 0.03 | 0.16 | 0.17 | 0.04 | 0.04 | 0.20 | 0.56 | 1.00 | 0.24 | 0.24 | 0.15 | 0.38 | 0.05 |
| 1154 | -0.46 | -0.45 | -0.50 | -0.58 | 0.07 | 0.03 | 0.42 | 0.01 | 0.11 | -0.41 | -0.20 | -0.31 | 0.15 | 0.00 | -0.05 | -0.24 | 0.18 | 0.19 | 0.09 | 0.17 | -0.01 | 0.16 | 0.24 | 1.00 | 0.98 | 0.23 | 0.18 | 0.18 |
| 1517 | -0.45 | -0.42 | -0.52 | -0.61 | 0.15 | 0.09 | 0.47 | 0.03 | 0.12 | -0.39 | -0.13 | -0.32 | 0.13 | 0.09 | -0.02 | -0.31 | 0.13 | 0.11 | -0.02 | 0.06 | 0.03 | 0.17 | 0.24 | 0.98 | 1.00 | 0.28 | 0.20 | 0.21 |
| MA | -0.49 | -0.41 | -0.17 | -0.41 | -0.21 | -0.31 | -0.07 | -0.41 | -0.20 | -0.29 | 0.03 | 0.03 | 0.08 | 0.20 | -0.11 | -0.12 | -0.31 | -0.21 | -0.27 | -0.31 | 0.26 | 0.23 | 0.15 | 0.23 | 0.28 | 1.00 | 0.42 | 0.43 |
| Gr | -0.49 | -0.50 | -0.23 | -0.30 | -0.26 | -0.35 | -0.03 | -0.24 | -0.16 | -0.38 | -0.20 | -0.26 | -0.02 | -0.01 | -0.30 | 0.03 | -0.06 | 0.06 | 0.05 | 0.04 | 0.16 | 0.78 | 0.38 | 0.18 | 0.20 | 0.42 | 1.00 | 0.82 |
| PL | -0.51 | -0.44 | -0.10 | -0.22 | -0.33 | -0.46 | 0.08 | -0.33 | -0.12 | -0.42 | -0.24 | -0.18 | 0.12 | 0.07 | -0.13 | 0.08 | -0.02 | 0.09 | 0.11 | 0.12 | 0.10 | 0.39 | 0.05 | 0.18 | 0.21 | 0.43 | 0.82 | 1.00 |

**Table S8.** Correlation coefficient values for Raman data and clinical information heat map in 3^rd^ trimester associated with Figure 5f.

|  | SoP | 1154 | 1517 | 835 | 1420 | 848 | 956 | 1584 | 1551 | 757 | 1206 | 1017 | 1031 | 1306 | 1316 | 1340 | GA | 1243 | 1171 | 898 | 940 | PL | Gr | Pa | MA | HoP | 1672 | Ch. Hyp | BMI | Cu. Hyp. |
| --- | --- | --- | --- | --- | --- | --- | --- | --- | --- | --- | --- | --- | --- | --- | --- | --- | --- | --- | --- | --- | --- | --- | --- | --- | --- | --- | --- | --- | --- | --- |
| SoP | 1.00 | 0.56 | 0.53 | 0.24 | 0.34 | 0.45 | 0.55 | 0.43 | -0.50 | -0.24 | -0.43 | -0.44 | -0.38 | -0.29 | -0.35 | -0.33 | -0.49 | -0.57 | 0.01 | 0.28 | 0.35 | -0.01 | -0.08 | -0.17 | 0.11 | 0.15 | -0.29 | -0.06 | -0.17 | 0.05 |
| 1154 | 0.56 | 1.00 | 0.98 | 0.09 | -0.07 | 0.11 | 0.29 | 0.10 | -0.62 | -0.35 | -0.57 | -0.37 | -0.59 | -0.09 | -0.16 | -0.19 | -0.29 | -0.60 | -0.05 | 0.16 | 0.16 | -0.09 | -0.09 | -0.04 | 0.11 | 0.18 | -0.06 | -0.08 | -0.39 | -0.10 |
| 1517 | 0.53 | 0.98 | 1.00 | 0.07 | -0.13 | 0.08 | 0.22 | 0.08 | -0.59 | -0.37 | -0.60 | -0.46 | -0.68 | -0.05 | -0.15 | -0.19 | -0.29 | -0.62 | -0.12 | 0.17 | 0.11 | -0.07 | -0.03 | 0.02 | 0.16 | 0.20 | -0.03 | -0.11 | -0.42 | -0.10 |
| 835 | 0.24 | 0.09 | 0.07 | 1.00 | 0.48 | 0.65 | 0.59 | 0.35 | 0.00 | 0.36 | -0.04 | 0.09 | 0.11 | -0.34 | -0.30 | -0.20 | 0.09 | -0.24 | -0.02 | 0.43 | 0.42 | -0.14 | -0.21 | -0.21 | -0.21 | -0.24 | -0.29 | -0.04 | -0.09 | 0.02 |
| 1420 | 0.34 | -0.07 | -0.13 | 0.48 | 1.00 | 0.61 | 0.79 | 0.41 | -0.10 | 0.01 | -0.15 | 0.15 | 0.21 | -0.72 | -0.57 | -0.39 | -0.04 | -0.36 | -0.06 | 0.16 | 0.42 | -0.10 | -0.16 | -0.15 | 0.02 | -0.18 | -0.29 | -0.17 | -0.10 | -0.02 |
| 848 | 0.45 | 0.11 | 0.08 | 0.65 | 0.61 | 1.00 | 0.81 | 0.56 | -0.03 | 0.33 | -0.03 | 0.07 | 0.16 | -0.32 | -0.35 | -0.28 | -0.02 | -0.27 | 0.09 | 0.62 | 0.59 | -0.05 | -0.10 | -0.13 | -0.22 | -0.18 | -0.34 | -0.10 | -0.09 | 0.01 |
| 956 | 0.55 | 0.29 | 0.22 | 0.59 | 0.79 | 0.81 | 1.00 | 0.60 | -0.24 | 0.07 | -0.15 | 0.12 | 0.16 | -0.44 | -0.32 | -0.17 | -0.10 | -0.45 | 0.22 | 0.60 | 0.79 | -0.14 | -0.24 | -0.22 | -0.11 | -0.09 | -0.37 | -0.20 | -0.21 | -0.04 |
| 1584 | 0.43 | 0.10 | 0.08 | 0.35 | 0.41 | 0.56 | 0.60 | 1.00 | 0.11 | 0.12 | -0.05 | 0.02 | 0.19 | -0.28 | -0.22 | -0.11 | -0.01 | -0.28 | 0.49 | 0.48 | 0.55 | 0.05 | 0.01 | -0.05 | -0.01 | 0.11 | -0.25 | -0.20 | -0.05 | -0.09 |
| 1551 | -0.50 | -0.62 | -0.59 | 0.00 | -0.10 | -0.03 | -0.24 | 0.11 | 1.00 | 0.63 | 0.69 | 0.49 | 0.59 | 0.06 | 0.25 | 0.36 | 0.54 | 0.74 | 0.25 | 0.05 | -0.03 | -0.02 | 0.08 | 0.11 | -0.08 | -0.22 | 0.17 | -0.16 | 0.36 | 0.12 |
| 757 | -0.24 | -0.35 | -0.37 | 0.36 | 0.01 | 0.33 | 0.07 | 0.12 | 0.63 | 1.00 | 0.69 | 0.45 | 0.54 | 0.15 | 0.30 | 0.36 | 0.46 | 0.61 | 0.32 | 0.27 | 0.23 | -0.10 | 0.02 | 0.10 | -0.17 | -0.22 | 0.34 | -0.01 | 0.23 | 0.19 |
| 1206 | -0.43 | -0.57 | -0.60 | -0.04 | -0.15 | -0.03 | -0.15 | -0.05 | 0.69 | 0.69 | 1.00 | 0.64 | 0.76 | 0.47 | 0.71 | 0.77 | 0.37 | 0.85 | 0.58 | 0.29 | 0.28 | -0.12 | -0.01 | 0.10 | -0.12 | -0.06 | 0.24 | -0.07 | 0.42 | 0.19 |
| 1017 | -0.44 | -0.37 | -0.46 | 0.09 | 0.15 | 0.07 | 0.12 | 0.02 | 0.49 | 0.45 | 0.64 | 1.00 | 0.89 | 0.12 | 0.36 | 0.48 | 0.48 | 0.49 | 0.57 | 0.21 | 0.30 | -0.09 | -0.11 | -0.04 | -0.27 | -0.22 | 0.07 | -0.14 | 0.18 | 0.06 |
| 1031 | -0.38 | -0.59 | -0.68 | 0.11 | 0.21 | 0.16 | 0.16 | 0.19 | 0.59 | 0.54 | 0.76 | 0.89 | 1.00 | 0.13 | 0.37 | 0.49 | 0.46 | 0.56 | 0.65 | 0.29 | 0.40 | -0.06 | -0.06 | -0.03 | -0.22 | -0.18 | 0.03 | -0.12 | 0.30 | 0.06 |
| 1306 | -0.29 | -0.09 | -0.05 | -0.34 | -0.72 | -0.32 | -0.44 | -0.28 | 0.06 | 0.15 | 0.47 | 0.12 | 0.13 | 1.00 | 0.88 | 0.69 | 0.02 | 0.44 | 0.41 | 0.26 | 0.08 | 0.03 | 0.15 | 0.19 | -0.09 | 0.13 | 0.15 | 0.09 | 0.17 | 0.07 |
| 1316 | -0.35 | -0.16 | -0.15 | -0.30 | -0.57 | -0.35 | -0.32 | -0.22 | 0.25 | 0.30 | 0.71 | 0.36 | 0.37 | 0.88 | 1.00 | 0.94 | 0.17 | 0.60 | 0.57 | 0.31 | 0.26 | -0.05 | 0.03 | 0.12 | -0.05 | 0.10 | 0.21 | 0.01 | 0.27 | 0.17 |
| 1340 | -0.33 | -0.19 | -0.19 | -0.20 | -0.39 | -0.28 | -0.17 | -0.11 | 0.36 | 0.36 | 0.77 | 0.48 | 0.49 | 0.69 | 0.94 | 1.00 | 0.26 | 0.60 | 0.63 | 0.35 | 0.37 | -0.10 | -0.03 | 0.07 | -0.05 | 0.06 | 0.22 | -0.05 | 0.27 | 0.19 |
| GA | -0.49 | -0.29 | -0.29 | 0.09 | -0.04 | -0.02 | -0.10 | -0.01 | 0.54 | 0.46 | 0.37 | 0.48 | 0.46 | 0.02 | 0.17 | 0.26 | 1.00 | 0.39 | 0.16 | 0.01 | 0.00 | 0.02 | 0.06 | 0.10 | -0.09 | -0.22 | 0.18 | 0.13 | 0.14 | 0.01 |
| 1243 | -0.57 | -0.60 | -0.62 | -0.24 | -0.36 | -0.27 | -0.45 | -0.28 | 0.74 | 0.61 | 0.85 | 0.49 | 0.56 | 0.44 | 0.60 | 0.60 | 0.39 | 1.00 | 0.28 | -0.09 | -0.12 | -0.10 | 0.02 | 0.13 | -0.12 | -0.09 | 0.24 | 0.10 | 0.44 | 0.16 |
| 1171 | 0.01 | -0.05 | -0.12 | -0.02 | -0.06 | 0.09 | 0.22 | 0.49 | 0.25 | 0.32 | 0.58 | 0.57 | 0.65 | 0.41 | 0.57 | 0.63 | 0.16 | 0.28 | 1.00 | 0.53 | 0.60 | -0.06 | 0.02 | 0.09 | -0.14 | 0.18 | 0.07 | -0.19 | 0.16 | 0.00 |
| 898 | 0.28 | 0.16 | 0.17 | 0.43 | 0.16 | 0.62 | 0.60 | 0.48 | 0.05 | 0.27 | 0.29 | 0.21 | 0.29 | 0.26 | 0.31 | 0.35 | 0.01 | -0.09 | 0.53 | 1.00 | 0.85 | -0.14 | -0.15 | -0.15 | -0.24 | 0.00 | -0.20 | -0.24 | -0.03 | -0.04 |
| 940 | 0.35 | 0.16 | 0.11 | 0.42 | 0.42 | 0.59 | 0.79 | 0.55 | -0.03 | 0.23 | 0.28 | 0.30 | 0.40 | 0.08 | 0.26 | 0.37 | 0.00 | -0.12 | 0.60 | 0.85 | 1.00 | -0.16 | -0.21 | -0.17 | -0.11 | 0.03 | -0.25 | -0.23 | -0.01 | 0.03 |
| PL | -0.01 | -0.09 | -0.07 | -0.14 | -0.10 | -0.05 | -0.14 | 0.05 | -0.02 | -0.10 | -0.12 | -0.09 | -0.06 | 0.03 | -0.05 | -0.10 | 0.02 | -0.10 | -0.06 | -0.14 | -0.16 | 1.00 | 0.82 | 0.36 | 0.19 | 0.08 | 0.00 | 0.17 | 0.06 | 0.26 |
| Gr | -0.08 | -0.09 | -0.03 | -0.21 | -0.16 | -0.10 | -0.24 | 0.01 | 0.08 | 0.02 | -0.01 | -0.11 | -0.06 | 0.15 | 0.03 | -0.03 | 0.06 | 0.02 | 0.02 | -0.15 | -0.21 | 0.82 | 1.00 | 0.81 | 0.24 | 0.30 | 0.11 | 0.08 | -0.01 | 0.10 |
| Pa | -0.17 | -0.04 | 0.02 | -0.21 | -0.15 | -0.13 | -0.22 | -0.05 | 0.11 | 0.10 | 0.10 | -0.04 | -0.03 | 0.19 | 0.12 | 0.07 | 0.10 | 0.13 | 0.09 | -0.15 | -0.17 | 0.36 | 0.81 | 1.00 | 0.18 | 0.45 | 0.13 | 0.01 | -0.03 | -0.10 |
| MA | 0.11 | 0.11 | 0.16 | -0.21 | 0.02 | -0.22 | -0.11 | -0.01 | -0.08 | -0.17 | -0.12 | -0.27 | -0.22 | -0.09 | -0.05 | -0.05 | -0.09 | -0.12 | -0.14 | -0.24 | -0.11 | 0.19 | 0.24 | 0.18 | 1.00 | 0.17 | 0.07 | 0.11 | -0.06 | 0.05 |
| HoP | 0.15 | 0.18 | 0.20 | -0.24 | -0.18 | -0.18 | -0.09 | 0.11 | -0.22 | -0.22 | -0.06 | -0.22 | -0.18 | 0.13 | 0.10 | 0.06 | -0.22 | -0.09 | 0.18 | 0.00 | 0.03 | 0.08 | 0.30 | 0.45 | 0.17 | 1.00 | -0.01 | -0.01 | 0.04 | -0.15 |
| 1672 | -0.29 | -0.06 | -0.03 | -0.29 | -0.29 | -0.34 | -0.37 | -0.25 | 0.17 | 0.34 | 0.24 | 0.07 | 0.03 | 0.15 | 0.21 | 0.22 | 0.18 | 0.24 | 0.07 | -0.20 | -0.25 | 0.00 | 0.11 | 0.13 | 0.07 | -0.01 | 1.00 | -0.11 | -0.12 | -0.01 |
| Ch.Hyp. | -0.06 | -0.08 | -0.11 | -0.04 | -0.17 | -0.10 | -0.20 | -0.20 | -0.16 | -0.01 | -0.07 | -0.14 | -0.12 | 0.09 | 0.01 | -0.05 | 0.13 | 0.10 | -0.19 | -0.24 | -0.23 | 0.17 | 0.08 | 0.01 | 0.11 | -0.01 | -0.11 | 1.00 | 0.42 | 0.03 |
| BMI | -0.17 | -0.39 | -0.42 | -0.09 | -0.10 | -0.09 | -0.21 | -0.05 | 0.36 | 0.23 | 0.42 | 0.18 | 0.30 | 0.17 | 0.27 | 0.27 | 0.14 | 0.44 | 0.16 | -0.03 | -0.01 | 0.06 | -0.01 | -0.03 | -0.06 | 0.04 | -0.12 | 0.42 | 1.00 | 0.42 |
| Cu. Hyp | 0.05 | -0.10 | -0.10 | 0.02 | -0.02 | 0.01 | -0.04 | -0.09 | 0.12 | 0.19 | 0.19 | 0.06 | 0.06 | 0.07 | 0.17 | 0.19 | 0.01 | 0.16 | 0.00 | -0.04 | 0.03 | 0.26 | 0.10 | -0.10 | 0.05 | -0.15 | -0.01 | 0.03 | 0.42 | 1.00 |

**Table S9**. Raman metabolites associated with each KEGG metabolic pathway shown in Figure 4.

| **KEGG pathway** | **Metabolites** |
| --- | --- |
| Alanine, aspartate and glutamate metabolism | Glutamic acid, citric acid |
| Aminoacyl-tRNA biosynthesis | Histidine, phenylalanine, arginine, glycine, methionine, threonine, tryptophan, tyrosine, glutamic acid |
| Arginine and proline metabolism | Glutamic acid, arginine |
| Arginine biosynthesis | Glutamic acid, arginine |
| Beta-Alanine metabolism | Histidine |
| Biosynthesis of unsaturated fatty acids | Palmitic acid |
| Citrate cycle (TCA cycle) | Citric acid |
| Cysteine and methionine metabolism | Methionine |
| D-Glutamine and D-glutamate metabolism | Glutamic acid |
| Fatty acid biosynthesis | Palmitic acid, myristic acid, lauric acid |
| Fatty acid degradation | Palmitic acid |
| Fatty acid elongation | Palmitic acid |
| Glutathione metabolism | Glutamic acid, glycine |
| Glycine, serine and threonine metabolism | Glycine, threonine |
| Glyoxylate and dicarboxylate metabolism | Citric acid, glutamic acid, glycine |
| Histidine metabolism | Glutamic acid, histidine |
| Nitrogen metabolism | Glutamic acid |
| Phenylalanine metabolism | Phenylalanine, tyrosine |
| Phenylalanine, tyrosine, and tryptophan biosynthesis | Phenylalanine, tyrosine |
| Primary bile acid biosynthesis | Glycine, cholesterol |
| Pyrimidine metabolism | Thymine |
| Retinol metabolism | β-carotene |
| Starch and sucrose metabolism | Glucose, trehalose |
| Steroid biosynthesis | Cholesterol |
| Steroid hormone biosynthesis | Cholesterol |
| Tryptophan metabolism | Tryptophan |
| Tyrosine metabolism | Tyrosine |
| Valine, leucine and isoleucine biosynthesis | Threonine |

**Table S10**. Raman peaks and their statistical significance in every trimester.

| **Wavenumber (cm^-1^)** | **Significance in 1^st^ trimester** | **Significance in 2^nd^ trimester** | **Significance in 3^rd^ trimester** |
| --- | --- | --- | --- |
| 681 | 0.0224 | 0.967 | 0.202 |
| 701 | 0.0335 | 0.497 | 0.095 |
| 718 | 0.110 | 0.573 | 0.182 |
| 743 | 0.825 | 0.399 | 0.637 |
| 757 | 0.135 | 0.076 | 0.304 |
| 805 | 0.576 | 0.275 | 0.0086 |
| 835 | 0.173 | 0.014 | 0.187 |
| 848 | 0.054 | 0.163 | 0.0011 |
| 876 | 0.0015 | 0.082 | 0.493 |
| 898 | 0.0009 | 0.844 | 0.0321 |
| 940 | 0.0003 | 0.188 | 0.0029 |
| 956 | 0.0190 | 0.004 | 0.000004 |
| 987 | 0.0014 | 0.157 | 0.504 |
| 1002 | 0.164 | 0.387 | 0.793 |
| 1017 | 0.430 | 0.0047 | 0.003 |
| 1031 | 0.544 | 0.009 | 0.0093 |
| 1056 | 0.405 | 0.713 | 0.967 |
| 1078 | 0.269 | 0.804 | 0.755 |
| 1105 | 0.337 | 0.557 | 0.417 |
| 1126 | 0.316 | 0.530 | 0.609 |
| 1154 | 0.0006 | 0.0120 | 0.000158 |
| 1171 | 0.683 | 0.283 | 0.578 |
| 1206 | 0.229 | 0.000001 | 0.0007 |
| 1243 | 0.052 | 0.000001 | 0.000006 |
| 1265 | 0.516 | 0.0013 | 0.148 |
| 1303 | 0.108 | 0.145 | 0.206 |
| 1317 | 0.171 | 0.0107 | 0.0106 |
| 1340 | 0.169 | 0.047 | 0.024 |
| 1355 | 0.020 | 0.060 | 0.115 |
| 1420 | 0.460 | 0.0119 | 0.0072 |
| 1445 | Normalized | Normalized | Normalized |
| 1517 | 0.0006 | 0.0039 | 0.000024 |
| 1551 | 0.0089 | 0.0005 | 0.00019 |
| 1584 | 0.347 | 0.196 | 0.00015 |
| 1606 | 0.501 | 0.420 | 0.153 |
| 1617 | 0.112 | 0.128 | 0.951 |
| 1657 | 0.0257 | 0.038 | 0.700 |
| 1672 | 0.731 | 0.041 | 0.0178 |

**
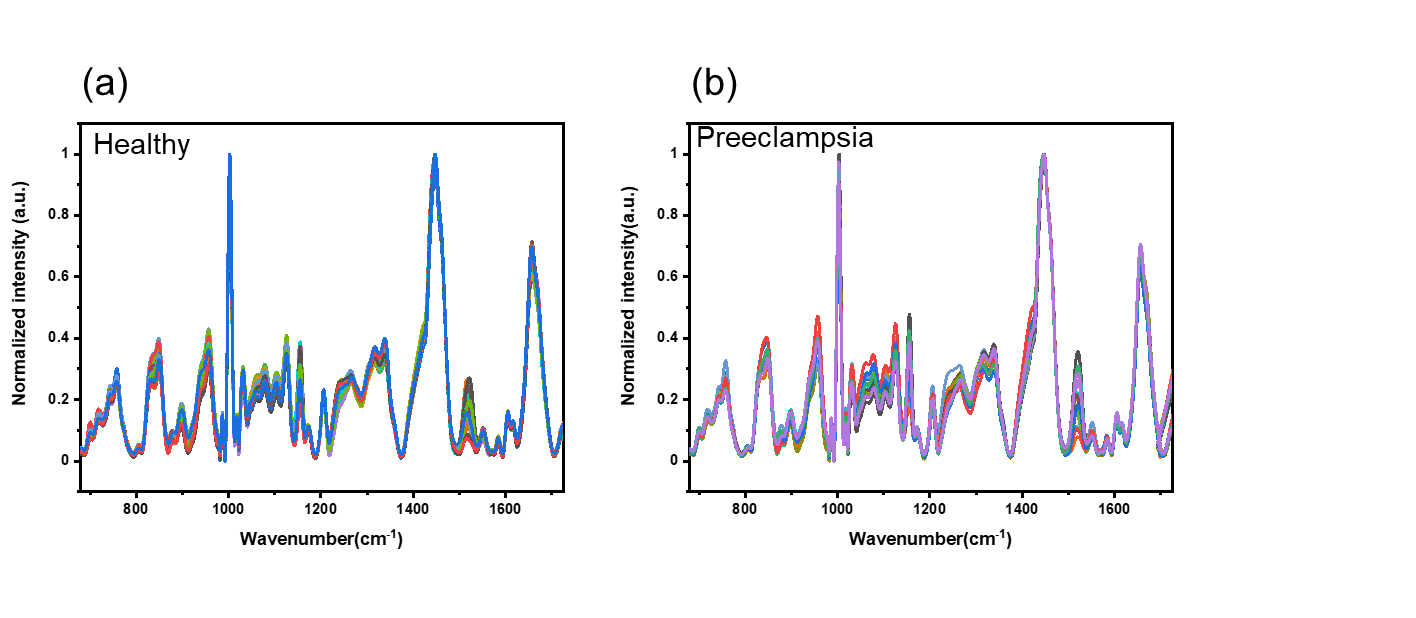

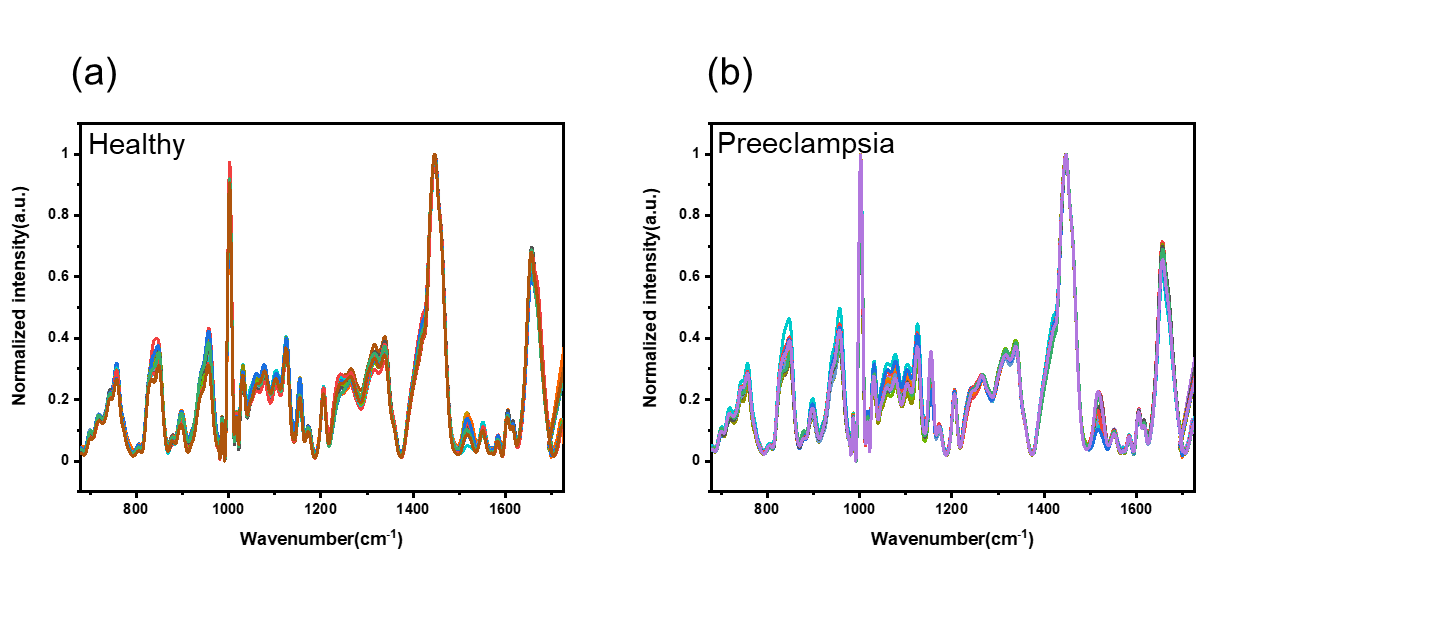
Figure S1.** (a) The Raman spectra of n=23 healthy samples tested in the 1^st^ trimester. (b) The Raman spectra of n=22 preeclamptic samples tested in the 1^st^ trimester.

**Figure S2.** (a) The Raman spectra of n=25 healthy samples tested in the 2^nd^ trimester. (b) The Raman spectra of n=20 preeclamptic samples tested in the 2^nd^ trimester.


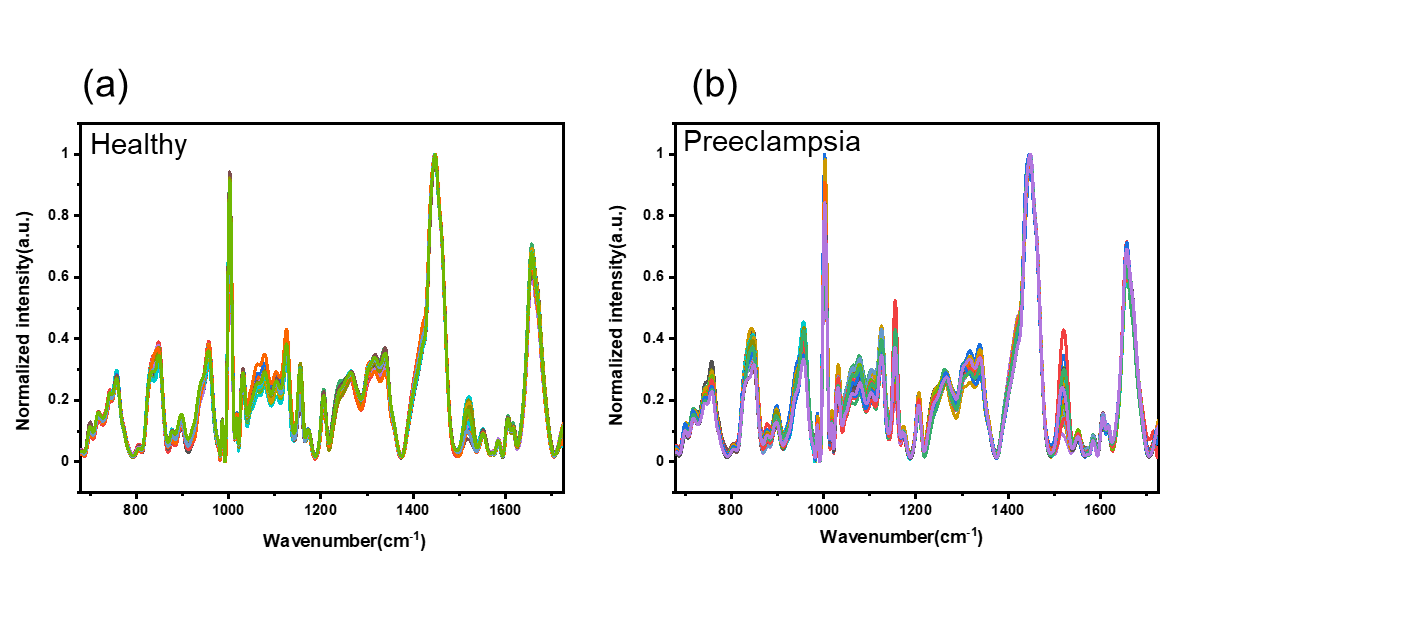


**Figure S3.** (a) The Raman spectra of n=24 healthy samples tested in the 3^rd^ trimester. (b) The Raman spectra of n=29 preeclamptic samples tested in the 3^rd^ trimester.


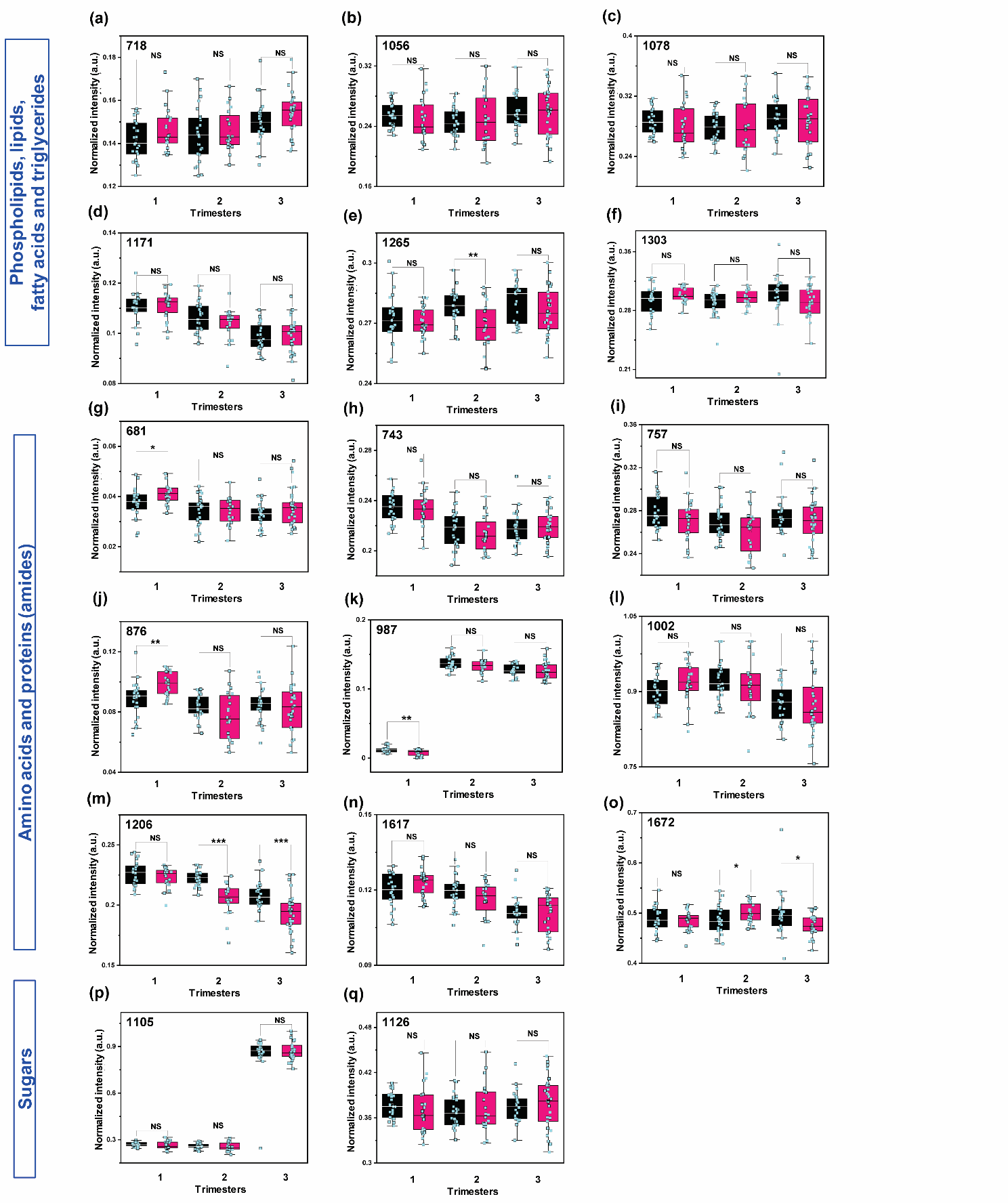


**Figure S4.** Box plots of remaining Raman peaks of healthy (**black**) and Preeclampsia (**pink**) patient cohorts in all 3 trimesters. Listed metabolites are phosphatidylcholine (718 cm^-1^), lipids (1078, 1063 cm^-1^), saturated long-chain fatty acids(1171 cm^-1^), unsaturated lipids and fatty acids (1265 cm^-1^), triglycerides(1303 cm^-1^), methionine (681 cm^-1^), thymine (743 cm^-1^), tryptophan (757 cm^-1^), glutamic acid (876 cm^-1^), arginine (987 cm^-1^), phenylalanine (1002 cm^-1^), general amino acids (1206 cm^-1^), tyrosine (1617 cm^-1^), amide I (1672 cm^-1^), mannose/trehalose (1105 cm^-1^) and glucose (1126 cm^-1^). Statistical significance between each cohort is noted with stars. Here, * represents a *p* *value* < 0.05, ** represents a *p value* < 0.01 and, *** represents a p value< 0.001 and NS stands for not significant.


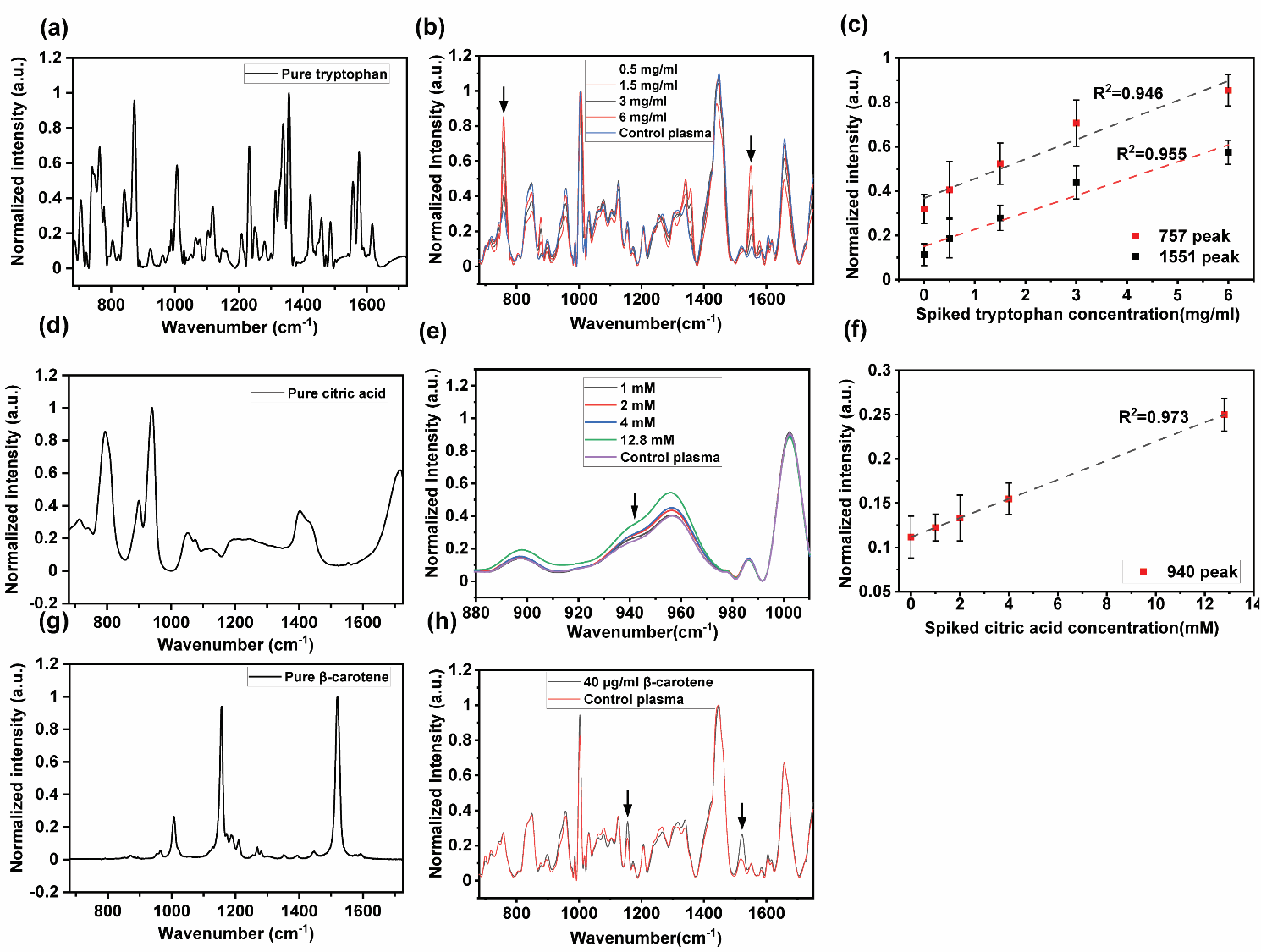


**Figure S5**. Spiking metabolites in human plasma to confirm selected Raman peaks. (a) Raman spectra of pure tryptophan (6 mg/mL) dissolved in water and then drop-dried on a CaF_2_ disk. (b) Normalized Raman spectra of multiple concentrations of tryptophan (0.5, 1.5, 3, 6 mg/ml) spiked into plasma sample from one of the healthy pregnant cohort. The tryptophan peaks are indicated with an arrow. (c) Both tryptophan Raman peak values at 757 and 1551 cm^-1^ plotted against the concentration of spiked tryptophan show a strong linear correlation. (d) Raman spectra of pure citric acid (2.45 mg/mL) dissolved in water and then drop-dried on a CaF_2_ disk. (e) Normalized Raman spectra of multiple concentrations of citric acid (1, 2, 4, 12.8 mM) spiked into control plasma sample from one of the healthy pregnant cohort. The spectra is cropped so the change in the 940 cm^-1^ shoulder peak assigend to citric acid is clearly visible. (f) Raman peak values at 940 cm^-1^ plotted against the concentration of spiked citric acid show a linear correlation. Note: in this paper 955 cm^-1^ peak is assigned to myristic acid, a long chain fatty acid, which has substantially stronger vibrations and higher Raman cross-section than citric acid, a small molecule. Fatty acids are abundant in plasma and very bright in Raman spectra. (g) Raman spectra of pure β-carotene (1 mg/mL) dissolved in ethanol and then drop-dried on a CaF_2_ disk. (h) Raman spectra of β-carotene (40 µg/mL) spiked into plasma sample from one of the healthy pregnant cohort. The β-carotene peaks are indicated with an arrow; the peaks at 1154 and 1517 cm^-1^ increase with spiking. Note that β-carotene is highly hydrophobic and therefore it is important to identify a suitable solvent that will solubilize β-carotene and enable homogenous dispersion in plasma.
